# Hierarchical Supramolecular Aggregation of Molecular Nanoparticles for Granular Materials with Ultra High‐Speed Impact‐Resistance

**DOI:** 10.1002/advs.202405285

**Published:** 2024-07-24

**Authors:** Xin Zhou, Jia‐Fu Yin, Cong Chen, Yanjie Chi, Jiadong Chen, Wei Liu‐Fu, Junsheng Yang, Shuchang Long, Liqun Tang, Xiaohu Yao, Panchao Yin

**Affiliations:** ^1^ State Key Laboratory of Luminescent Materials and Devices & School of Emergent Soft Matter Guangdong Basic Research Center of Excellence for Energy and Information Polymer Materials South China University of Technology Guangzhou 510640 China; ^2^ School of Civil Engineering and Transportation State Key Laboratory of Subtropical Building Science South China University of Technology Guangzhou 510640 China

**Keywords:** impact resistance, molecular granular materials, molecular nanoparticle, relaxation dynamics, supramolecular materials

## Abstract

The high‐speed impact‐resistanct materials are of great significance while their development is hindered by the intrinsic tradeoff between mechanical strength and energy dissipation capability. Herein, the new chemical system of molecular granular material (MGM) is developed for the design of impact‐resistant materials from the supramolecular complexation of sub‐nm molecular clusters (MCs) and hyper‐branched polyelectrolytes. Their hierarchical aggregation provides the origin of the decoupling of mechanical strengths and structural relaxation dynamics. The MCs’ intrinsic fast dynamics afford excellent high‐speed impact‐resistance, up to 5600 s^−1^ impact in a typical split‐Hopkinson pressure bar test while only tiny boundary cracks can be observed even under 7200 s^−1^ impact. The high loadings of MCs and their hierarchical aggregates provide high‐density sacrificial bonding for the effective dissipation of the impact energy, enabling the protection of fragile devices from the direct impact of over 200 m s^−1^ bullet. Moreover, the MGMs can be conveniently processed into protective coatings or films with promising recyclability due to the supramolecular interaction feature. The research not only reveals the unique relaxation dynamics and mechanical properties of MGMs in comparison with polymers and colloids, but also develops new chemical systems for the fabrication of high‐speed impact‐resistant materials.

## Graphical abstract

Molecular granular material is proposed for the design of impact resistance materials from the supramolecular complexation of sub‐nm molecular clusters and hyperbranched polymers with the decoupling of mechanical strengths and the fast relaxation dynamics of sub‐nm particles. The fast dynamics afford excellent high‐speed impact resistance while the hierarchical aggregates provide high‐density, multi‐mode bonding for effective dissipation of the impact energy.

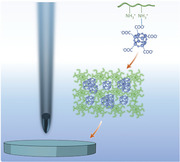

## 1 Introduction

Materials with superior impact resistance are key in protecting the structural and functional integrity of bio‐tissues, organs, and functional devices from external impacts, including projectile and shock wave impacts.^[^ ^1^ ^]^ Polymer elastomers,^[^ ^2^ ^]^ granular materials,^[^ ^3^ ^]^ aerosols^[^ ^4^ ^]^ and nanocomposites of layer assemblies^[^ ^5^ ^]^ have been under intense research for impact‐resistant materials and several have been commercialized in the sportswear and manufacturing industry. However, protection from high‐speed impact is still challenging while it is critical for the automobile, aviation, and aerospace industries. Especially, shock waves that travel at extremely high velocities (several thousand m·s^−1^) can bring devastating damage to human brains, lungs, and other organs while effective resistant materials for shock waves are still rare.^[^ ^6^ ^]^ The general mechanism for impact resistance is the absorption of impact energy by the protective materials through their shape deformation, the breakage of physical/chemical bonding, and/or the friction among the structural units.^[^ ^2^, ^7^ ^]^ The promising chemical systems require the simultaneous realization of fast structural relaxation dynamics that are matchable to fast impact speed and high energy densities of the corresponding structural units that can fully dissipate the amount of impact energies.^[^ ^2b,c^ ^]^ Unfortunately, the tradeoff between the relaxation dynamics rate and cohesive energy density seems to govern most of the chemical systems, e.g. polymers.^[^ ^8^ ^]^ Strong association among the basic structural units is required for high cohesive energy density, e.g. the friction of colloid nanoparticles (NPs) in granular materials^[^ ^9^ ^]^ and the aggregates of dynamic bonding in elastomers,^[^ ^7^, ^10^ ^]^ which, however, leads to the arresting effect and slowing down of relaxation dynamics of certain structural units. Therefore, the invention of new chemical systems that are different from polymers and colloid NPs could be the solution to break the above‐mentioned general tradeoff for the effective resistance of high‐speed impacts.

**Figure 1:**
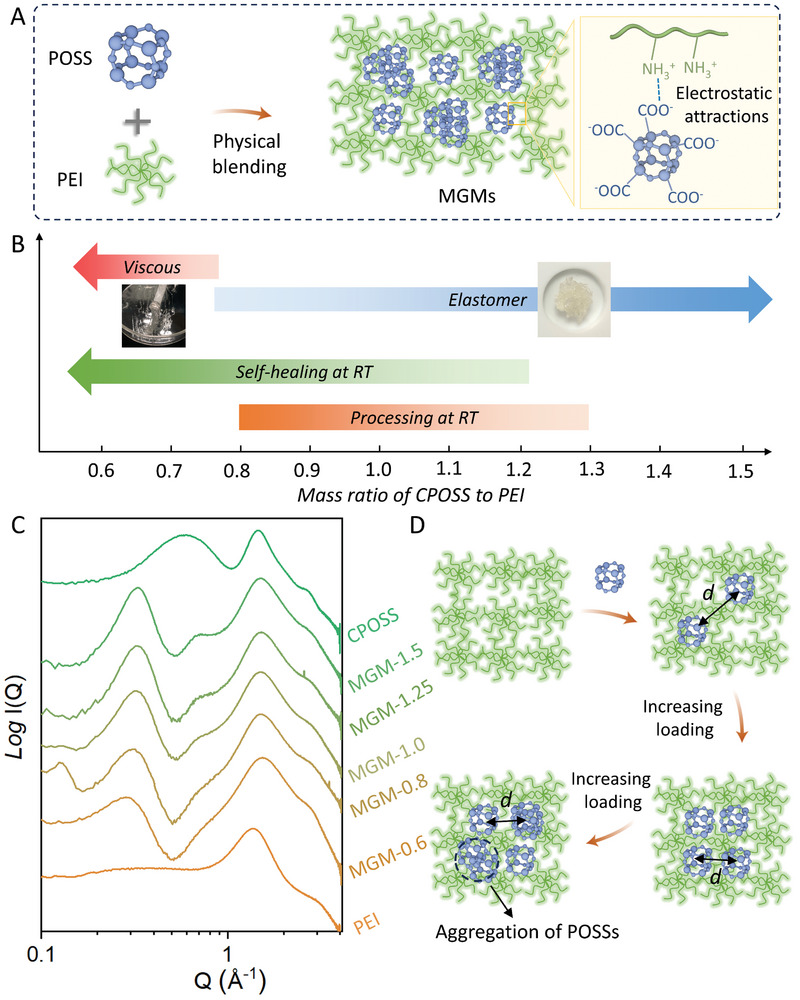
Design strategy and structural characterizations for MGMs. A) Schematical illustration for the preparation of MGMs via facial physical blending approaches. B) Mechanical properties regulation through tuning mass ratio of CPOSS to PEI. C) SAXS data of CPOSS‐PEI composites with different loading of CPOSS (MGMs‐n, n refers to the mass ratio of CPOSS to PEI). MGM‐1.0 was selected for subsequent characterization and testing. D) Schematic illustration shows the hierarchical aggregation of CPOSS in a physical crosslinking network with increasing loading of CPOSS.

The emergent materials with their characteristic sizes at the sub‐nm scale have attracted general interest in condensed matter physics, chemical science, and material science due to their distinct physical/chemical properties from small molecules and colloid NP systems.^[^ ^11^ ^]^ The sub‐nm particles show unique, fast structural relaxation dynamics with energy levels close to that of thermal fluctuation.^[^ ^12^ ^]^ Meanwhile, the material system possesses an extremely high surface‐to‐volume ratio, and the structural relaxation of surface structures can be dominated and even decoupled from the overall diffusive dynamics of the particles. The aggregation of the sub‐nm particles and the disruption of their cooperative dynamics by the topologies of their assemblies lead to the hindered diffusive dynamics, but resumed fast surface structure relaxation dynamics.^[^ ^12a‐c^ ^]^ This provides potential solutions for the general tradeoff of materials’ strength and toughness. Herein, molecular granular materials (MGMs) are fabricated from the supramolecular complexation and close packing of sub‐nm polyhedral oligomeric silsesquioxane (POSS) units and hyperbranched polyethyleneimine (PEI) at different loadings for the studies of their potential for the resistance of high‐speed mechanical and shock wave impact (**Figure** **1**a). The hierarchical structure from the complexation of PEI and POSS and the aggregation of POSS clusters are tuned via the loading of POSS, which are probed using small angle X‐ray scattering (SAXS) techniques.^[^ ^13^ ^]^ The mechanical properties are examined for their correlation with the aggregation of POSS clusters. The selected POSS‐PEI composites are further tested against falling ball impact test, split‐Hopkinson pressure bar (SHPB) measurements, and air gun projectile tests to quantify their capacity for the dissipation of high‐speed impact energy. Their excellent impact‐resistant capacities are further confirmed for the protection of fragile devices from impact force.

## 2 Results and Discussion

### 2.1 Material Design and Structural Characterization

POSS cluster carrying 8 carboxylic acid groups (CPOSS) is synthesized and complexed with hyper‐branched PEI at different mass ratios from 0.6:1 to 1.5:1 (Figure 1a; Figures S1–S6, Supporting Information). The CPOSS interacts with PEI via both ionic and hydrogen bonding interaction^[^ ^14^ ^]^ (Figure S7, Supporting Information) and the physical appearance can be broadly tuned from viscous liquid to elastomers (Figure 1b; Figure S8, Supporting Information), providing a feasible approach to optimized and versatile impact resistant capability. The SEM and SEM‐EDS mapping show that CPOSS and PEI are uniformly distributed in the MGMs (Figure S9, Supporting Information). The hierarchical supramolecular structures can be deciphered from SAXS studies whereas both the form factor of CPOSS and the structure factor corresponding to the aggregation of CPOSS can be observed (Figure 1c; Figure S10, Supporting Information). The microphase formation, characterized by the scattering peak at 0.67 to 0.74 Å^−1^ as the inter‐phase distance, can be observed at 0.8 loadings of CPOSS and above. Meanwhile, the inter‐POSS distance interpreted from the scattering peak at 0.29 to 0.32 Å^−1^ range decreases and then stabilizes at 0.32 Å^−1^ with the increasing of CPOSS loadings, suggesting the close packing of CPOSS in their aggregates (Figure 1d). The CPOSS prefers to interact with the amine groups of PEI and the sites are saturated with loadings of CPOSS at 0.8. The further increase of CPOSS loadings leads to the aggregation of CPOSS into micro‐phases, which act as additional crosslinking domains and well‐dispersed soft NPs for mechanical strengthening.^[^ ^15^ ^]^ Branched PEI and CPOSS with low molecular weights possess fast relaxation dynamics and act as soft phases to provide energy dissipation capability to the MGMs. Moreover, the noncovalent crosslinking between branched polymer chains and CPOSS favors the development of healable, recyclable polymeric nanocomposites with feasible processability^[^ ^16^ ^]^ (Figures S11–S13, Supporting Information). PEI demonstrates high affinity to water from a large number of amine groups and the residual water could facilitate the dynamic property of non‐covalent interactions and promote energy dissipation of MGMs.

**Figure 2:**
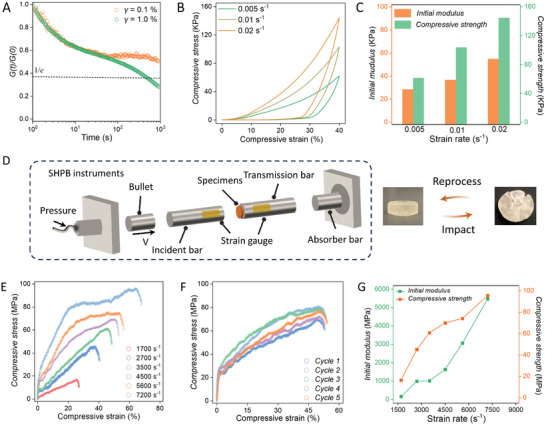
Mechanical performance evaluation for MGMs at different strain rates. A) Stress relaxation of MGMs at different strains. B) Compressive stress‐strain curves of MGMs under low strain rate loadings. C) Dependence of initial modulus and compressive strength on strain rate of MGMs. D) Schematic illustration of the SHPB system. It mainly consists of a bullet, an incident bar, and a transmission bar. The Inset picture is the disclike specimens with a thickness of 4 mm and a diameter of 10 mm, which can be facilely reprocessed after failure. E) Compressive stress‐strain curves of MGMs at different strain rates from SHPB tests. F) Compressive stress–strain curves of reprocessed MGMs under repeat impact at 5600 s^−1^ in SHPB tests. G) Initial modulus and compressive strength of MGMs from SHPB tests.

The relaxation dynamics of the MGMs should be fast according to their low glass transition temperature (*T_g_* = −19 °C for MGM‐1.0 with CPOSS/PEI mass ratio as 1.0), which contributes to the energy dissipation capability for fast impact as well as promising self‐healing properties and processability^[^ ^2b,c^ ^]^ (Figure S14, Supporting Information). Meanwhile, thanks to the high crosslinking density from the interaction between CPOSS and PEI and the aggregation of CPOSS, MGMs can maintain exceptional elasticity and mechanical properties even far above their *T_g_* (Figures S15 and S16, Supporting Information). MGMs are not significantly hygroscopic and their morphology and mechanical properties do not change significantly for at least six months in a conventional environment. Stress relaxation and creep experiments demonstrated that a physical crosslinking network can relax under the external disturbance of a higher rate, achieving energy dissipation under high‐rate destruction (**Figure** **2**a; Figure S17, Supporting Information). By virtue of high‐density crosslinking physical networks and CPOSS aggregated nanodomains, MGMs exhibited obvious strain hardening under a high loading rate (Figure 2b). With the improvement of compressive strength and initial modulus, the energy dissipation density of MGMs can be gradually enhanced, reflecting its potential application in impact resistance and energy dissipation under high‐rate destruction^[^ ^5^, ^7^ ^]^ (Figure 2c; Figure S18, Supporting Information).

### 2.2 Impact Resistance Performance

Under typical split‐Hopkinson pressure bar (SHPB) tests, the MGMs remain intact when strain rates are up to 5600 s^−1^ (Figure 2d,e; Figure S19, Supporting Information) and they only yield with limited boundary failure at 7200 s^−1^ with high energy dissipation capacity as 29.96 J g^−1^ or 42.55 J cm^−3^ (Table S1, Supporting Information). The damaged MGMs can be simply reprocessed and recycled, and no obvious decrease in the mechanical performances can be measured during five consecutive impacts at 5600 s^−1^ (Figure 2f; Figures S20 and S21, Supporting Information). The significant rate dependence of compressive strength and initial modulus can be observed in both low and high‐shear rate regions, suggesting the capability to effectively absorb mechanical energy under high‐speed impact^[^ ^5^, ^7^ ^]^ (Figure 2g). The unique strain‐dependent behavior affords MGMs with excellent processability at low strain rates and enhanced modulus/strength at high strain rates for impact resistance^[^ ^12a^ ^]^ (Figure S22, Supporting Information). Unlike conventional polymer‐based shear thickening materials, MGMs are intrinsically elastomers in the absence of shear loading. The strain‐hardening behavior of MGMs mainly originates from the physical network and the phase separation nanodomains.^[^ ^17^ ^]^ The impact resistance can also be confirmed in a typical air gun projectile test with a high energy dissipation capacity of 16.54 J g^−1^ or 23.48 J cm^−3^ (Figures S23–S26, Movies S1–S4 and Table S2, Supporting Information). MGMs’ energy dissipation ability mainly derives from the hierarchical aggregation structure, including fast relaxing CPOSS/PEI structural units, the synergistic movement of reversible physical cross‐linked networks formed by non‐covalent bonds, and the deformation of the aggregated domain of MCs^[^ ^2^, ^10^, ^12^, ^15^ ^]^ (Figure 1d). MGM can further be assembled into laminated glasses as the energy dissipation layer. The as‐assembled sandwich‐like glass specimen is capable of withstanding the impact by steel projectile (m = 5 g) with extremely high velocity (*ca*. 202.5 m s^−1^). The back glass plate can be fully protected without any micro‐cracks, verifying the exceptional impact‐resistant capacity of MGM layers (Figure S27, Supporting Information).

**Figure 3:**
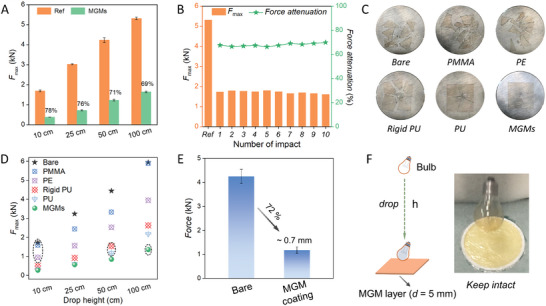
Impact protection performance of MGMs. A) Maximum value of force (*F*_max_) during the impact of MGMs determined from force‐time curves (Figure S29, Supporting Information). The mass of the steel ball is 0.225 kg and black text represents the percentage of force attenuation. Note that the force signals of reference condition (Ref) represent the direct impact of the ball toward the force sensor. B) Corresponding *F*_max_ and force attenuation for MGMs during ten repeated impacts from 100 cm. C) The impact protection performance of different cushions to glass. Note that the force signals of bare condition represent the direct impact of the ball toward the glass. D) Corresponding *F*_max_ of from 10 to 100 cm height. Black circles represent the initial height of glass breakage. E) The force attenuation of MGM coating from the impact of 50 cm. F) Snapshots of bulbs hitting MGMs that freely dropped from 2.2 m height.

The MGMs can be facilely processed into thin films and surface protective coatings and their impact resistance is confirmed by their protection of fragile devices against falling ball impact test (Figure S28, Supporting Information). The impacts are quantified by force sensors with and without (reference) the 3 mm protective film of MGMs, respectively (Figure S29, Supporting Information). The thin film can significantly attenuate the impact forces (*ca*. 70%) and prolong the buffer time (**Figure** **3**a). The force attenuation coefficient of MGMs shows no obvious decrease even after being repeatedly impacted ten times, suggesting superior and robust impact energy dissipation capability (Figure 3b; Figure S30–S32, Supporting Information). Rebound experiments^[^ ^18^ ^]^ further demonstrate the excellent energy dissipation capabilities of MGMs (Movie S5, Supporting Information). Impact protection tests are applied to commercial polymers, including polymethyl methacrylate (PMMA), polyethylene (PE), and polyurethane (PU), and interestingly, MGMs show the best energy dissipation capability at different dropping heights. To visualize the impact resistance performance, the 1 mm thin films of MGMs and polymers are applied to protect 1 mm ITO glass against falling balls^[^ ^5b^ ^]^ (Figure S33, Supporting Information). The MGMs can effectively prevent the glass from being damaged while the glass protected with polymeric materials shows various degrees of fragmentation (Figure 3c,d; Figures S34–S38, Supporting Information). Due to the feasible process, MGMs can be facilely processed as coatings on glass and exhibit excellent protection with a high force attenuation coefficient of 72% (Figure 3e; Figures S39 and S40, Supporting Information). Moreover, the MGM films can protect free‐falling beakers, light bulbs, and raw eggs, confirming their potential in the protection of functional materials and devices (Figure 3f; Figure S41 and Movies S6–S8, Supporting Information). The MGMs show a decoupled relationship between mechanical strengths and the fast relaxation dynamics of structural units. Therefore, the titled molecular granular material system demonstrates both promising mechanical strength and excellent resistance to high‐speed impacts. Generally, the applications of polymers for impact resistance materials are limited by the intrinsic tradeoffs between mechanical strengths and fast chain relaxation dynamics.

### 2.3 Microscopic Mechanism of the Impact Process

**Figure 4:**
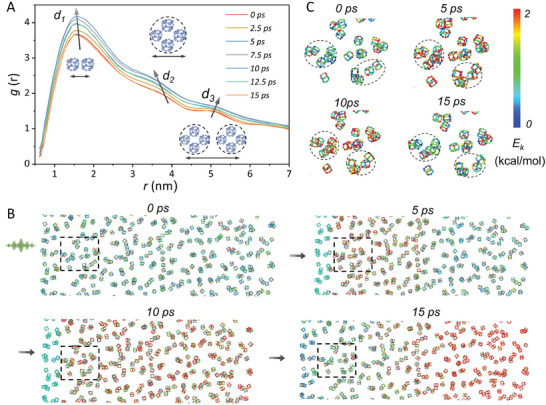
Nonequilibrium MD simulation of MGMs under shock‐wave. A) Evolution of the RDF plots of POSSs when exposed to a shock wave with the initial particle velocity of 0.5 km s^−1^. B) Snapshots of POSS during the shock wave impact, the colors of atoms refer to their kinetic energy (*E*_K_) levels. C) Local snapshots of POSS during the propagation of a shock wave. PEI molecules and surface‐grafted carboxylic acid groups are omitted for clarity.

To gain insight into the microscopic mechanism of the impact process, large‐scale molecular dynamics (MD) simulations under shock wave impact are performed. Suggesting from the evolution of radial distribution function (RDF), the characteristic distances, *d*_1_ (inter‐CPOSS distance) and *d*_2_ (microphase size) become smaller with the increasing of the shock wave loading time (from 0 to 10 ps), which confirms the enhancement of internal frictions between the primary granular units (**Figure** **4**a). An opposite trend is observed for *d*_3_ (the correlation distance of microphase), suggesting the deformation of micro‐domains of CPOSSs for energy adsorption. Once the shock wave is unloaded, the RDF curve quickly returns to the original state within 5 ps. This manifests the dominating role of fast structural relaxation dynamics in energy dissipations. As depicted in Figure 4b, the snapshots of spatially distributed POSSs inside MGM at different loading times are recorded. The kinetic energy (*E*_K_) distribution indicates that the shock wave propagation is related to the deformation of POSS agglomerated domains, which can be directly evidenced by the local snapshots (Figure 4c).

## 3 Conclusion

In summary, MGMs with both superior resistance for high‐speed impact and promising recyclability are designed by harnessing the hierarchical aggregation behaviors of sub‐nanoscaled molecular clusters. Comprehensive impact tests unambiguously demonstrate the superior impact resistance under extremely high strain rates of the MGMs. Quantitative analysis of the attenuation of impact force confirms that the protective capacity of MGMs outperformed many common polymeric materials. Systematical experiments combined with molecular dynamics simulations are exploited to reveal the underlying molecular origins. The rapid relaxation of structural units, fast exchange dynamics of the physically cross‐linked network, and deformation of agglomerated domain cooperatively contribute to the excellent energy dissipation capability. Our studies not only provide functional materials for high‐speed impact resistance, but also bring a new chemical system of the sub‐nm particles for the possible solution to the general tradeoffs of currently existing materials. Enriched functionalities that are beyond the performance of traditional materials are expected from the sub‐nm particle systems with appropriate molecular design.

## 4 Experimental Section

### General Materials

Octavinyloctasilasesquioxane (VPOSS, > 95.0%), Thioglycolic acid (> 98.0%), 2‐Hydroxy‐4′‐(2‐hydroxyethoxy)−2‐methylpropiophenone (Irgacure 2959, > 98.0%), Branched Polyethyleneimine (PEI, *M*_w_ = 1800 g mol^−1^, 99.0%) and Tetrahydrofuran (THF, 99.0%) were purchased from Shanghai Aladdin Biochemical Technology Co., Ltd. (Shanghai, China). The water used was deionized water.

### Synthesis of CPOSS

VPOSS (5 g, 7.89 mmol) and thioglycolic acid (6.17 g, 67.07 mmol) were dissolved in 50 mL THF. Then, Irgacure 2959 (141.5 mg, 0.63 mmol) was added to the solution and the solution was stirred for 5 min. The solution was irradiated under 365 nm UV light for 30 min. The solution was concentrated by rotary evaporation and precipitated into deionized water several times to remove the Irgacure 2959 and excess thioglycolic acid. The obtained pale yellow clear viscous liquid was dried in a vacuum oven for 48 h to afford the final product, CPOSS (9.8 g, 91%).

### Preparation of CPOSS/PEI Composites

CPOSS and PEI were dissolved in THF and deionized water to obtain 100 mg mL^−1^ solution respectively. Then, a certain amount of CPOSS solution was added to the PEI solution by setting the volume ratio of 0.6:1, 0.7:1, 0.8:1, 0.9:1, 1.0:1, 1.1:1, 1.25:1, and 1.5:1. The mixtures were stirred for 1 h at room temperature to ensure the homogenous mixing of CPOSS and PEI. The resultant mixtures were cast into petri dishes followed by drying the samples at room temperature for 24 h and at room temperature for 12 h in a vacuum oven. Accordingly, the as‐prepared cast CPOSS/PEI composites will be denoted as MGMs‐0.6, MGMs‐0.8, MGMs‐0.8, MGMs‐0.9, MGMs‐1.0, MGMs‐1.1, MGMs‐1.25, MGMs‐1.5, respectively. The resultant CPOSS/PEI composites are processed in the designed mold at RT for 5 min under the pressure of 5 MPa to obtain a specific shape for various subsequent tests.

### Characterization Methods

Nuclear magnetic resonance (NMR), Fourier transform infrared spectroscopy (FT‐IR), small angle X‐ray scattering (SAXS), differential scanning calorimetry (DSC), rheology, quasi‐static compression, split‐Hopkinson pressure bar (SHPB) experiments, air gun projectile impact, falling ball impact, and falling ball impact protection are employed for structural characterizations and mechanical property evaluations. Due to the limitation of this section, more details can be accessible in the attached supplemental information file.

### Statistical Analysis

The impact protection experimental data were presented as the mean ± SD (*n* = 3).

## Conflict of Interest

The authors declare no conflict of interest.

## Supporting information

Supporting Information

Supplemental Movie 1

Supplemental Movie 2

Supplemental Movie 3

Supplemental Movie 4

Supplemental Movie 5

Supplemental Movie 6

Supplemental Movie 7

Supplemental Movie 8

## Data Availability

The data that support the findings of this study are available from the corresponding author upon reasonable request.
