# Supplementary material for: Hierarchical Supramolecular Aggregation of Molecular Nanoparticles for Granular Materials with Ultra High‐Speed Impact‐Resistance
Source: Adv Sci (Weinh). 2024 Jul 24;11(36):2405285. doi: 10.1002/advs.202405285 (PMC11422806; doi:10.1002/advs.202405285)
Supplement: Supplementary file 1 — Supporting Information [file ADVS-11-2405285-s001.docx]

Supporting Information

Hierarchical Supramolecular Aggregation of Molecular Nanoparticles for Granular Materials with Ultra High-Speed Impact- Resistance

Xin Zhou,^†,1^ Jia-Fu Yin,^†,1^ Cong Chen,^2^ Jiadong Chen,^1^ Yanjie Chi,^1^ Wei Liu-Fu,^1^ Junsheng Yang,^1^ Shuchang Long,^2^ Liqun Tang,^2^ Xiaohu Yao,^2^ and Panchao Yin^1,*^

**1.** **Supplemental Movie Files**

**Movie S1:** Air gun projectile impact for MGMs with velocity of 58.6 m s^-1^.

**Movie S2:** Air gun projectile impact for MGMs with velocity of 59.4 m s^-1^.

**Movie S3:** Air gun projectile impact for MGMs with velocity of 52.6 m s^-1^.

**Movie S4:** Air gun projectile impact for MGMs with velocity of 53.6 m s^-1^.

**Movie S5:** Rebound rate experiment for MGMs from a height of 1 m.

**Movie S6:** MGMs protected beakers that freely falls from a height of 2.2 m.

**Movie S7:** MGMs protected light bulbs that freely falls from a height of 2.2 m.

**Movie S8:** MGMs protected raw eggs that freely falls from a height of 1.2 m.^[5b, 12a]^

**2. Instruments and characterizations**

**Nuclear Magnetic Resonance (NMR)**

Bruker AVANCE II 500 MHz spectrometer was used for NMR measurements. Before the test, the samples are dissolved in deuterated solvents, in which 0.1 v/v % tetramethylsilane (TMS) was employed as internal reference standard. The NMR data are recorded at 298 k, and the chemical shifts of our samples are therefore calibrated by referring to the characteristic signal of TMS (*δ* = 0, single peak).

**Matrix-assisted laser desorption/ionization-time of flight (MALDI-TOF) mass spectra**

MALDI-TOF measurements were carried out on a Waters HDMS Synapt XS system. In the sample preparation, *trans*-2-(3-(4-t-butyl-phenyl)-2-methyl-2-propenylidene) malononitrile (DCTB) matrix (20 mg/mL in THF) and cationizing agent NaTFA (10 mg/mL in THF) were mixed in the ratio of 10/1 (v/v). Samples were dissolved in THF at a concentration of 5 mg/ml.

**Scanning electron microscope (SEM)**

The SEM and SEM-EDS mapping were carried out on a JSM-7900F system. Thin film samples were adhered to conductive adhesive for testing.

**Fourier Transform Infrared Spectroscopy (FT-IR)**

Anhydrous KBr powder was shaped into thin pellet via pressure processing. The liquid like CPOSS was spread on the KBr pellet and then subjected to the Bruker Vector 33 FT-IR spectrometer to collect the FT-IR data. For elastomer-like MGM-1, no specific treatment to the sample was required. The sample was pressed against a high-refractive-index prism and the infrared data was recorded at attenuated total reflection (ATR) modes.

**Small Angle X-ray Scattering (SAXS)**

SAXS measurements were carried out at the beamlines 16B1 and 19U2 at Shanghai Radiation Facility (SSRF). The samples were sealed by Kapton films and fixed at a sample stage. The SAXS samples were irradiated by the high-flux X-ray for 10 s and the scatted X-ray was simultaneously probed by 2-dimensional (2D) detector (Pilatus 2M) behind the samples. The 2D SAXS pattern was reduced to 1D SAXS curves via Raw platform. The background scattering from the Kapton film and air should be subtracted. Generally, the scattering vector (*Q*) was defined as *Q* = 4π sinθ / λ, in which θ was half of the experimental scattering angle and λ was the wavelength of the incident X-ray beam. The resultant SAXS data cover abundant structure information spanning across a broad spatial range.

**Differential Scanning Calorimetry (DSC)**

Thermal analysis was carried out on a TA DSC 2500 instrument. The dried sample (2 - 5 mg) was sealed in aluminum crucible and empty aluminum crucible was used as reference. The sample was firstly heated from room temperature to 120 ^o^C to eliminate the undesirable thermal history. After that, thermal diagrams were thereby recorded with a heating/cooling rate of 10 K/min under nitrogen atmosphere. The as-afforded DSC cooling curve were applied to determine the glass transition temperature (*T_g_*) on TRIOS software package.

**Rheology**

The mechanical response of CPOSS/PEI composite under external force fields was studied by rheological measurements. A lab-standard Anton Paar MCR-302 rotational rheometer was employed for all the rheology tests. The sample was pre-shaped into circular thin film via pressure processing. For the rheological frequency sweep test, the sample was sheared with constant shearing strain (0.1 %) under sinusoidal frequency field (100 to 0.1 Hz) at room temperature. Temperature sweep test was performed from 0 to 60 ℃ under an angular frequency of 1 rad/s and a shear strain of 0.1%. Additionally, stress relaxation and creep test in the shear stress mode are also conducted to probe the relaxation behavior of CPOSS/PEI composites. The shear strain of stress relaxation was 0.1% or 1%, and the applied stress in creep test is 200 Pa, 500 Pa and 1000 Pa.

**Quasi-static compression test**

Quasi-static compressions were conducted on Instron 5965 tabletop testing system. Before the test, the MGM was processed into cylinder-like specimen with diameter of 10 mm and thickness of 4 mm. The as-prepared specimens were compressed with different strain rates to assess materials’ rate dependent mechanical properties.

**Split-Hopkinson pressure bar (SHPB) experiments**

A home-made split-Hopkinson pressure bar (SHPB) instrument was shown in **Scheme S1**, which basically consisted launching system, strike bar, incident bar, transmission bar, absorbed bar, and data acquisition system. Detail about the impact experiment was summarized as follow. The compressed air was abruptly released to accelerate the strike bar, and the so-caused collision between strike bar and incident bar gave rise to an elastic compressive wave. The strain rates of the compressive wave were positively correlated with the compressive air pressure. Herein, the strain rates in our SHPB experiments were varied to assess the materials’ rate-dependent mechanical properties. The compressive wave was then loaded on the MGM sample (cylinder specimen with diameter of 10 mm and thickness of 4 mm). The data acquisition system enable access to the information of the incident, reflect, and transmissive wave. The stress (σ(𝑡)), strain history (ɛ(𝑡)) and strain rate (ɛ̇(𝑡)) of the specimens can be describe as follows:

$$\begin{aligned} \sigma\left( t \right)=E_{o}\frac{A_{0}}{A}\varepsilon_{t}\left( t \right); \\ \dot{\varepsilon}\left( t \right)=-\frac{2C_{0}}{L}\varepsilon_{r}\left( t \right); \\ \varepsilon\left( t \right)=-\frac{2C_{0}}{L}\int_{0}^{t} \varepsilon_{r}\left( t \right)dt; \end{aligned}$$

where$E_{o}$ represented the elastic modulus of the incident bar; $A$ and $A_{0}$ represented the cross-section area of the specimen and the incident bar, respectively; $L$ was the thickness of the cylinder-shaped MGM sample; $C_{0}$ was the speed of the elastic wave; $\varepsilon_{r}\left( t \right)$ and $\varepsilon_{t}\left( t \right)$ referred to the reflected and transmitted strain pulse, respectively.


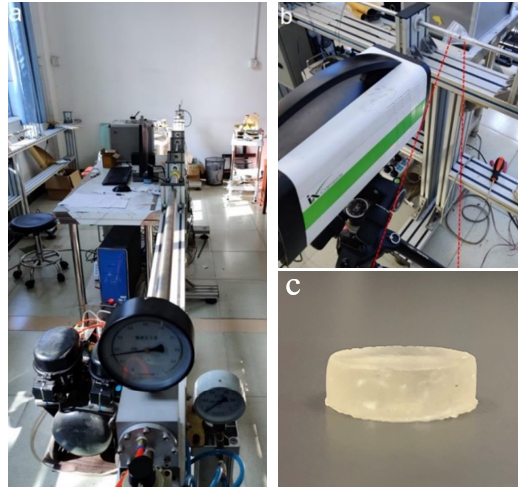


**Scheme S1**. **High speed impact tests on SHPB platform**. (a), (b) Overviews of the lab-standard SHPB platform used in our experiments. (b) High speed camera is exploited to track the failure process of MGM samples. (c) Digital photographs of the cylinder specimens used for the test.

**Air gun projectile impact test**


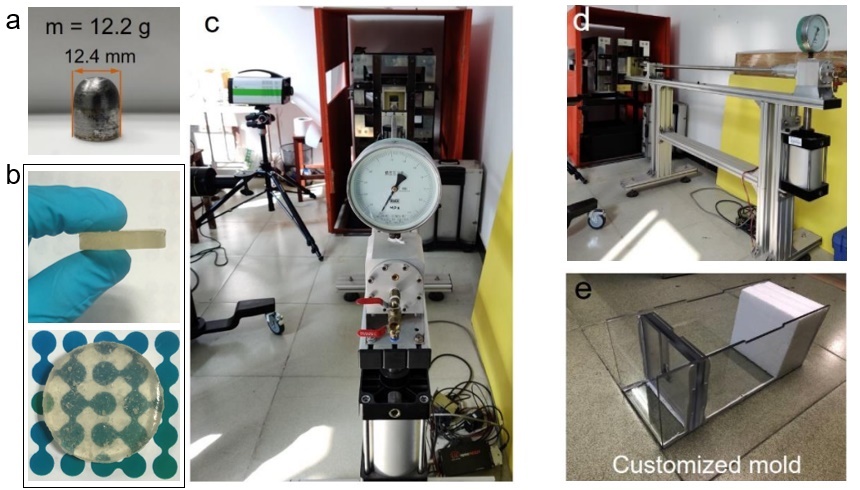


**Scheme S2**. **An overview of the air gun projectile impact study**. (a) and (b) The steel projectile and MGM specimen used in our experiments, respectively. (c) and (d) Digital photograph of our Lab-standard air gun projectile impact platform. (e) Customized mold we designed for the use of fixing and recycling the MGM samples.

Air gun projectile impact instruments^[12a]^ were shown in **Scheme S2**. The samples were firstly processed and shaped into disc-like specimen with a thickness of 6.2 mm and a diameter of 30 mm. Then, the specimen was clamped by the customized polycarbonate mold. Steel projectile (m = 12.2 g) was used for the impact test. The MGM samples were impacted by the projectile with different flying speeds. The impact process were recorded by the digital camera, more details please see in the attached video files.

**Falling ball impact test**

A falling ball equipment was employed for impact resistance. A steel ball weighing 0.255 kg was released from specified height to freely impact on MGM specimens placed on the surface of force sensor. Thickness of MGMs samples is 3 mm. The force signal was simultaneously recoded by the force sensor. For the control group, the steel ball was dropped from the same height and impact on the force sensor without the protection of MGM samples. The force signals from the MGM system and control groups were compared to assess the impact-resistant capacity of our MGM samples.

**Falling ball impact protection test**

The same falling instrument was used for the falling ball impact protection test. Indium-Tin Oxide (ITO) conductive glass is commercially available with a thickness of 1 mm. ITO glass was pre-coated with MGM layer via solution-casting. After the solvent evaporation, the thickness of the MGM layer was determined to be 1 mm. Falling ball tests were performed at different heights to evaluate the protection ability. The raw ITO glass and ITO glasses with PE, PU, PMMA coating were employed for control groups.

**Protection of fragile devices from falling impact force**

Free-falling beakers, light bulbs and raw eggs are protected by 5 mm MGMs sample. The falling heights of beakers, light bulbs and raw eggs are 2.2 m, 2.2 m and 1.2 m, respectively. The process of impact protection is recorded by camera.

**3. Supplemental figure**


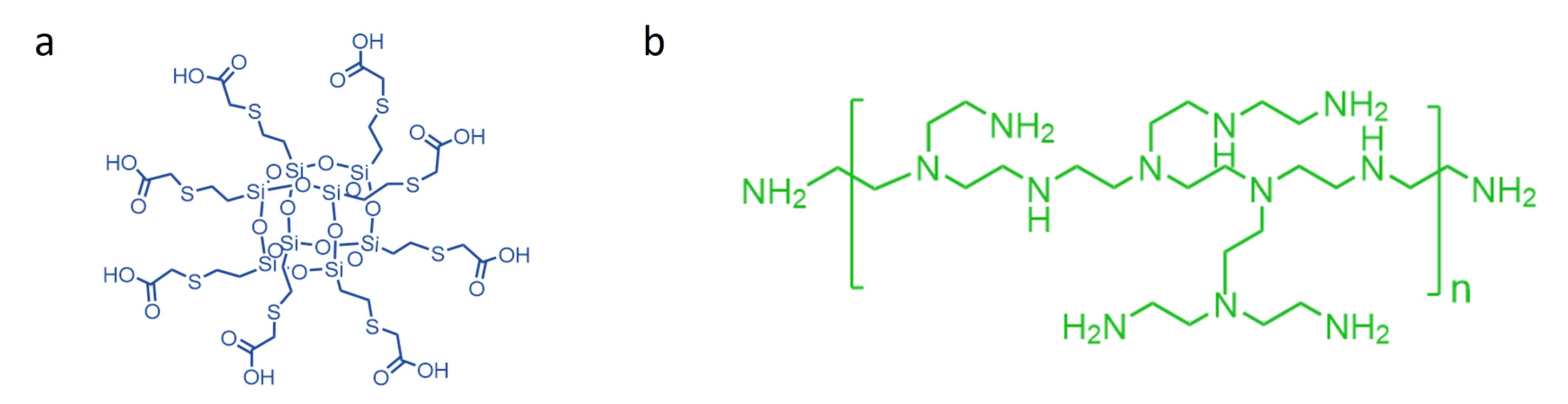


**Figure S1.** Chemical structures of CPOSS and PEI.


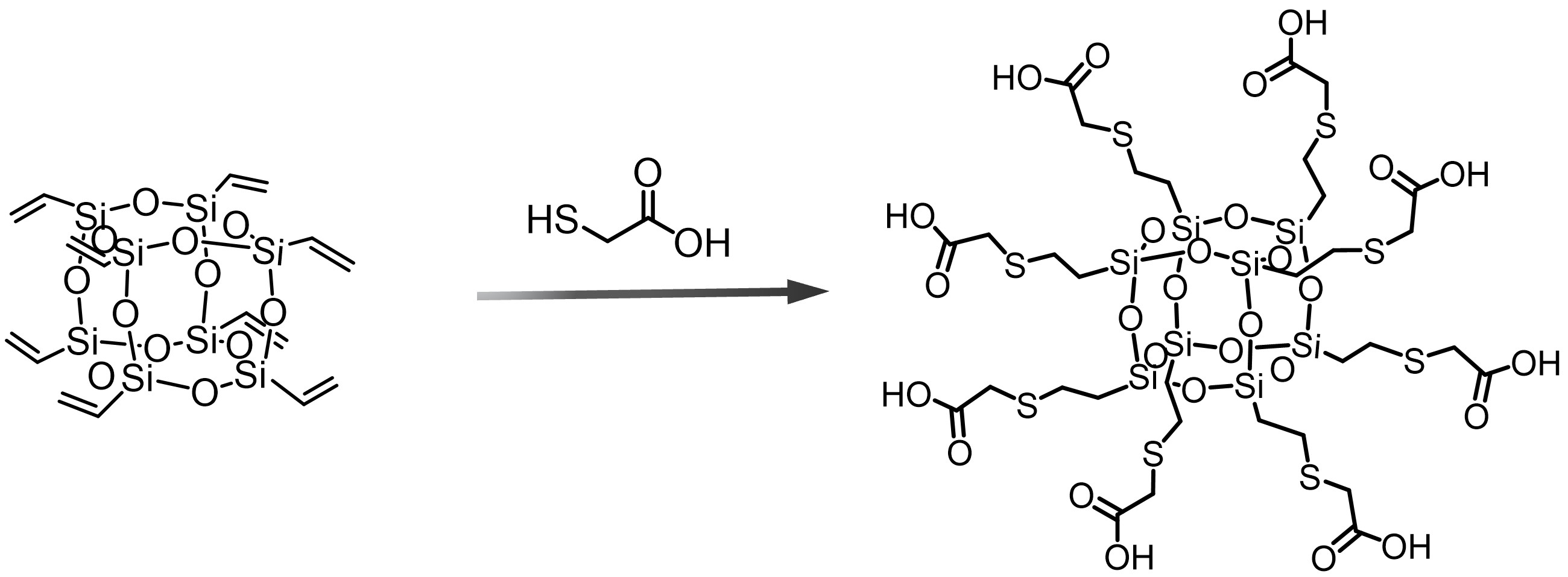


**Figure S2.** Synthesis route of CPOSS.


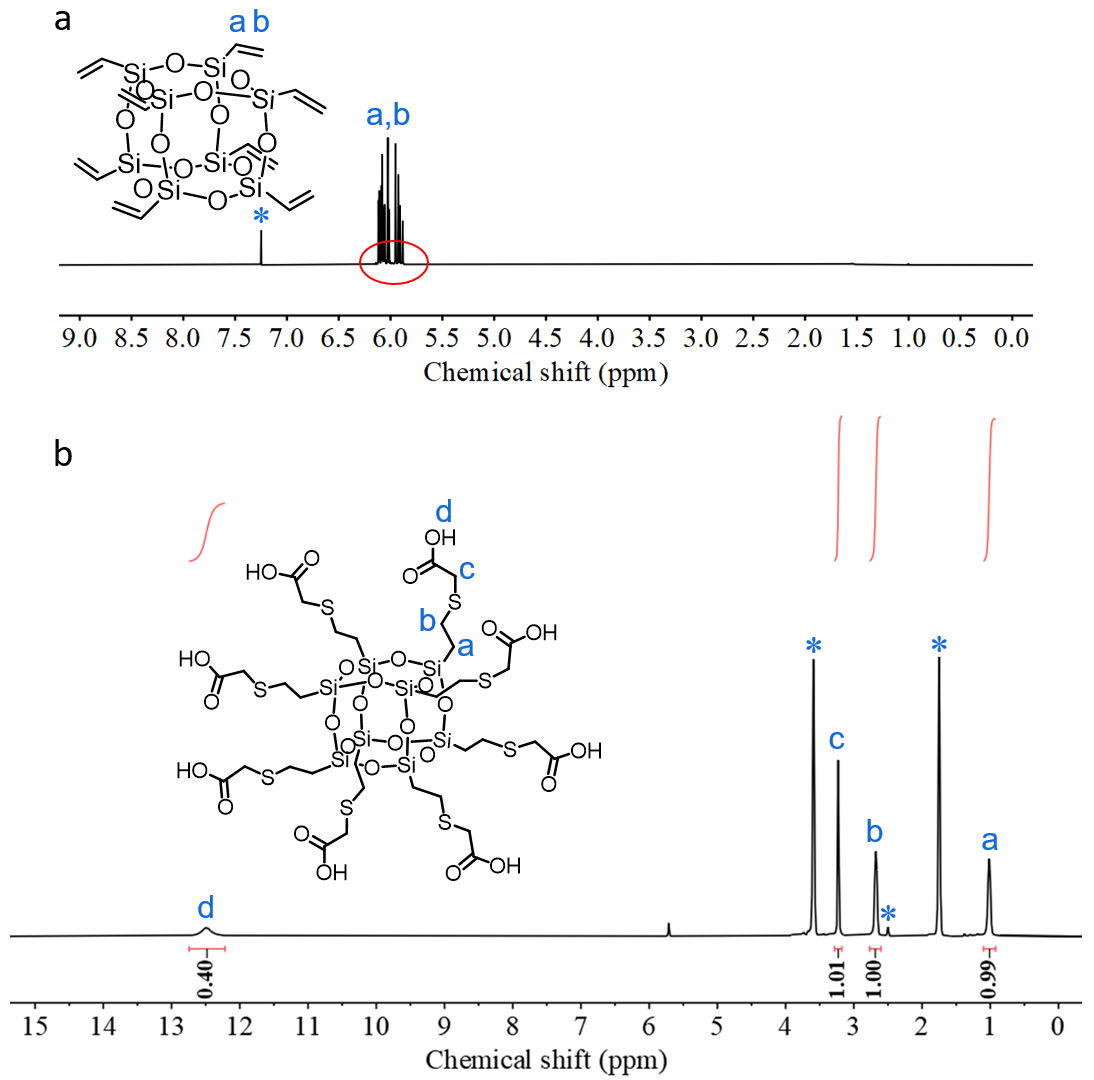


**Figure S3.** (a) ^1^H NMR spectra of VPOSS in CDCl_3_. (b) ^1^H NMR spectra of CPOSS in DMSO. The solvent peaks are labeled with *. The absence of the characteristic signal of -C=C at 6.0 ppm in the spectra indicated no -C=C residue and each CPOSS was successfully grafted with eight carboxyl groups.


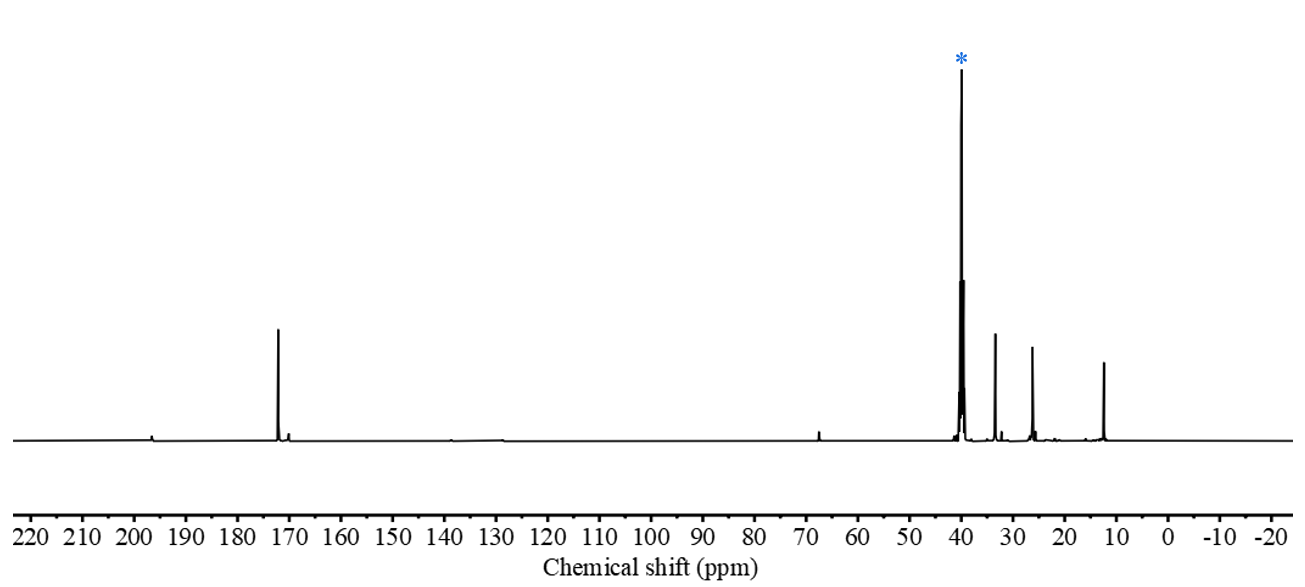


**Figure S4.** ^13^C NMR spectra of CPOSS in DMSO.


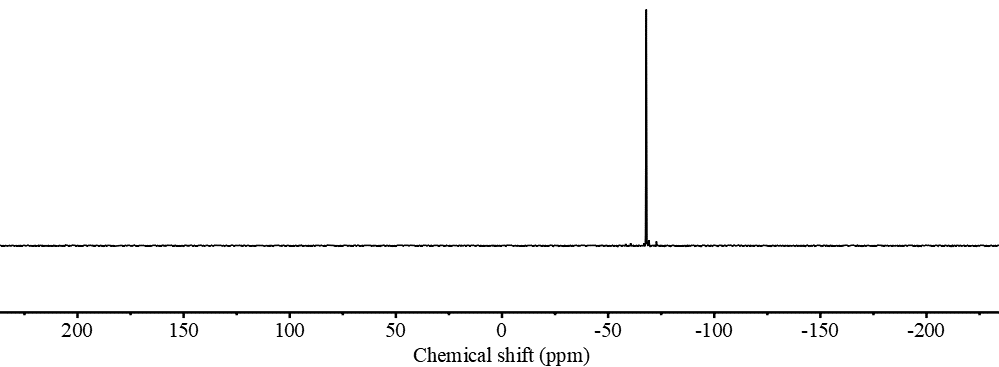


**Figure S5.** ^29^Si NMR spectra of CPOSS in DMSO.


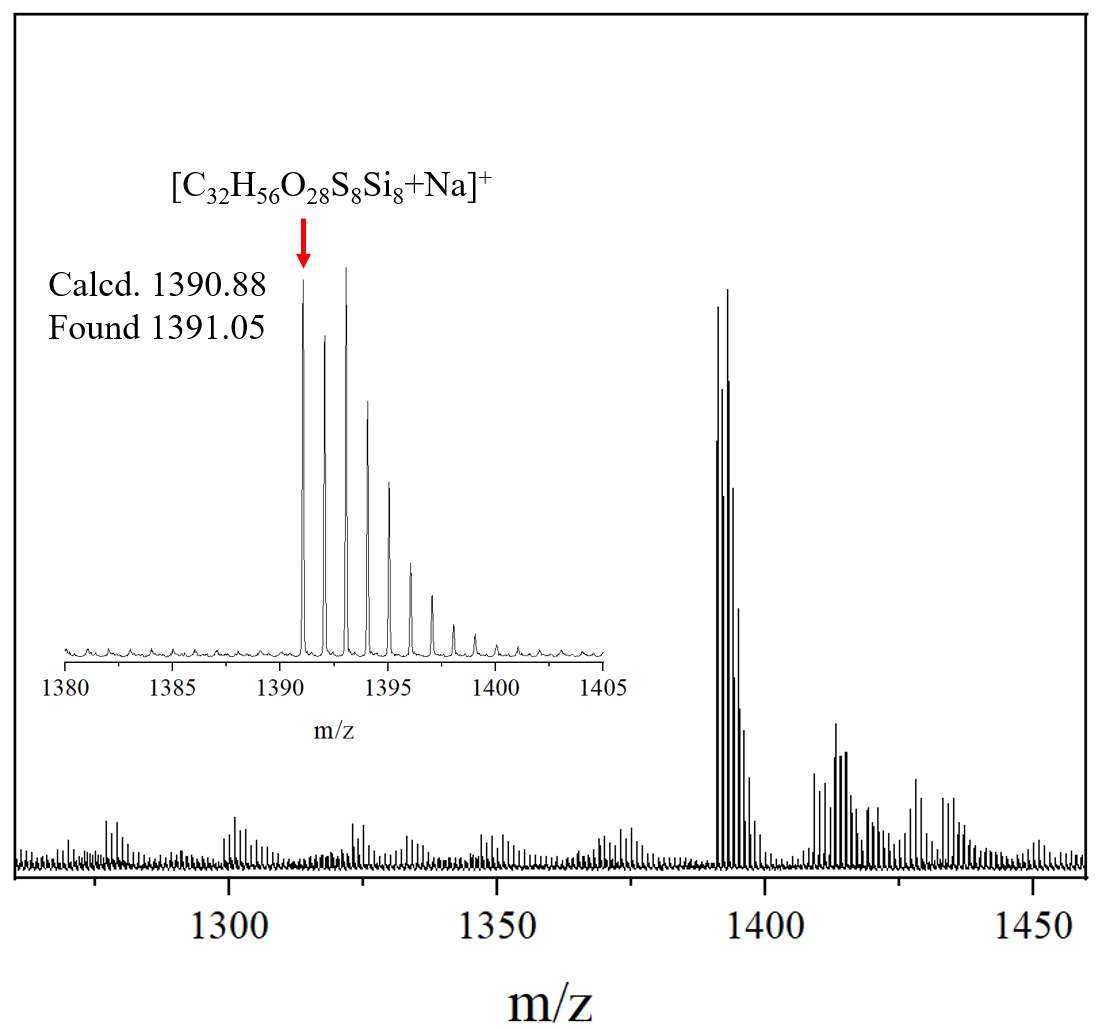


**Figure S6.** MALDI-TOF spectra of CPOSS.


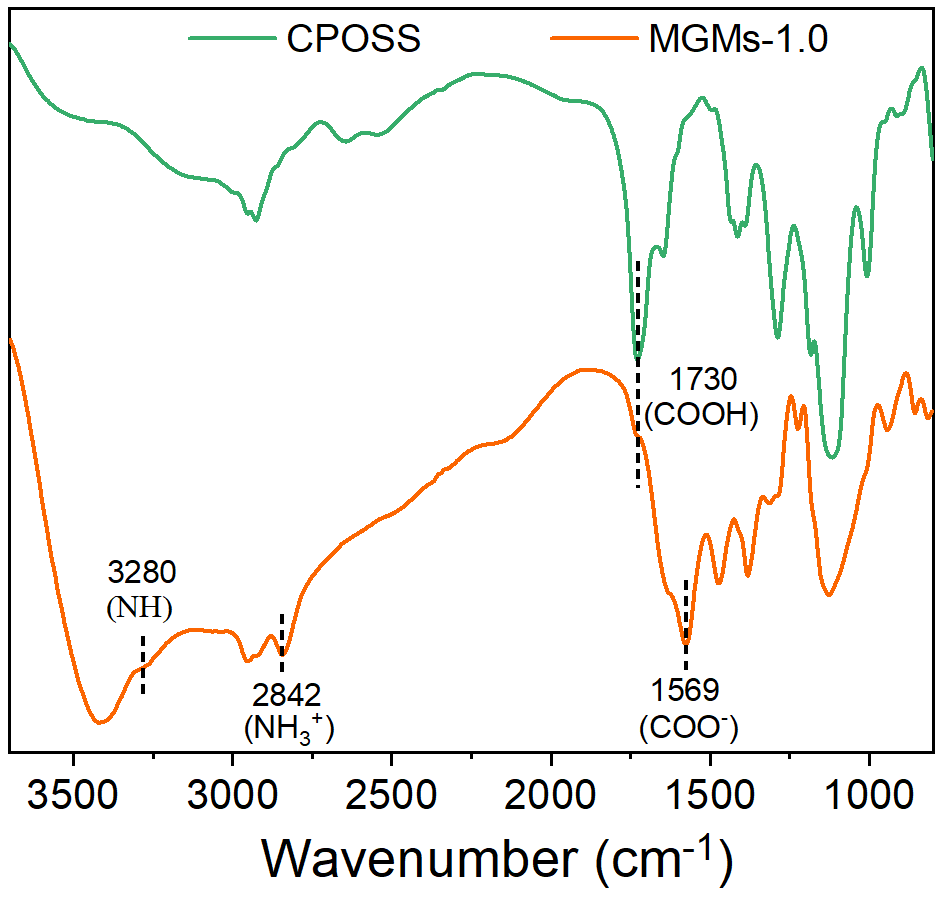


**Figure S7.** FT-IR spectra of CPOSS and MGMs. In the FTIR spectra, the characteristic IR adsorption bands for the -COOH, -COO-, N-H and NH_3_^+^ groups of MGMs are detected. Hydrogen bonding interactions can occur between the -COOH and N-H/NH_3_^+^ groups. Meanwhile, the NH_3_^+^ of PEI can have electrostatic interactions with -COO- groups of CPOSS. Therefore, PEI can have electrostatic and hydrogen-bonding interactions with CPOSS. The slight redshift of the band of Si-O-Si may be due to the formation of supramolecular interactions between POSS and PEI, resulting in an increase in the Si-O bond length.


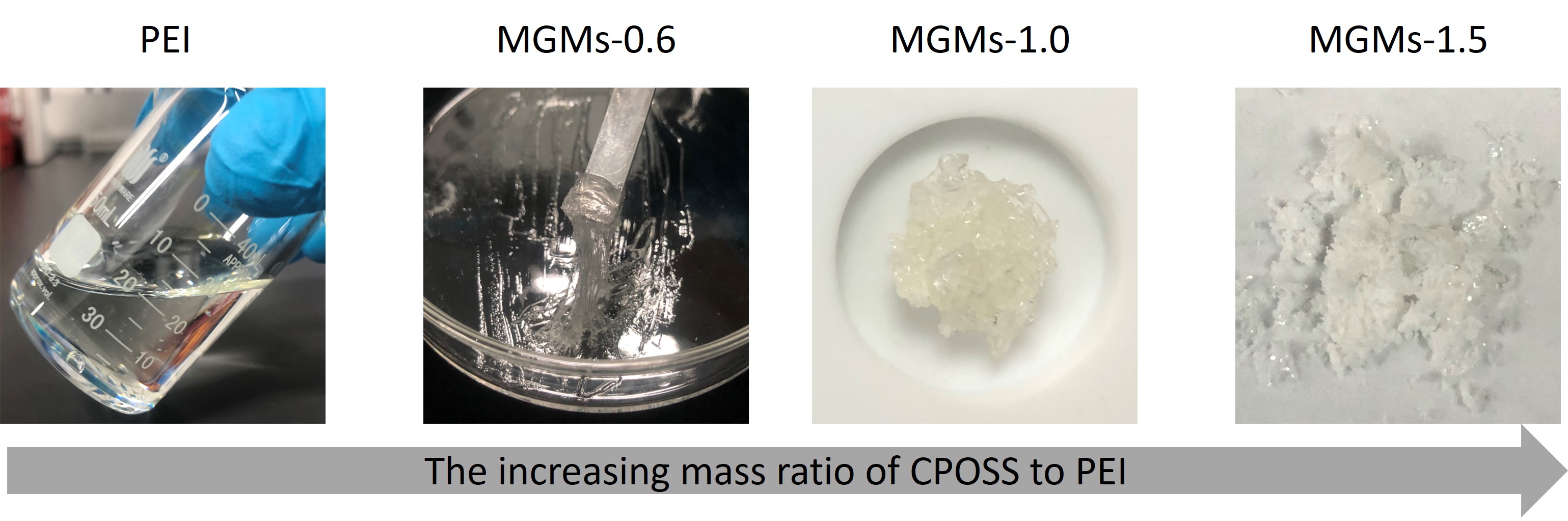


**Figure S8.** The regulation of mechanical properties of CPOSS-PEI composites with the increasing loading of CPOSS.


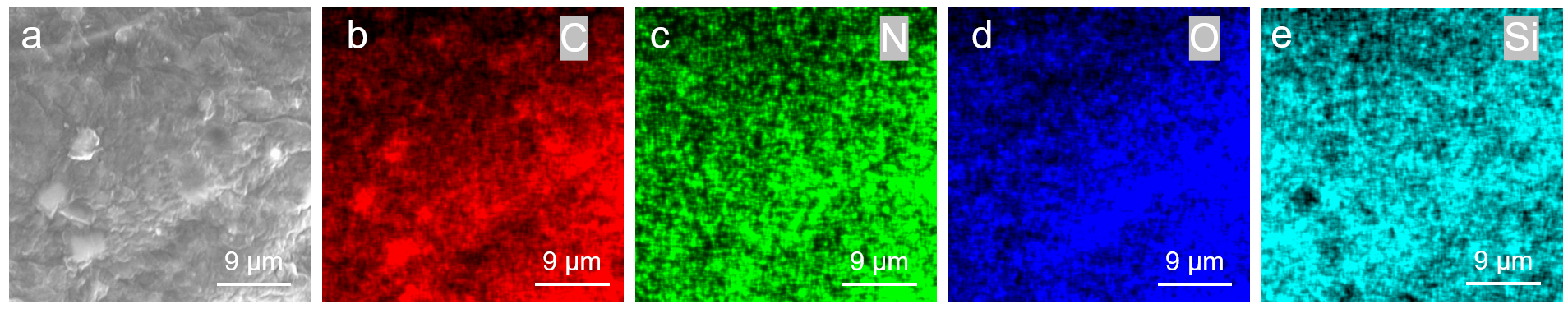


**Figure S9.** The SEM image (a) and SEM-EDS mapping (b-e) of MGM-1.0 sample.


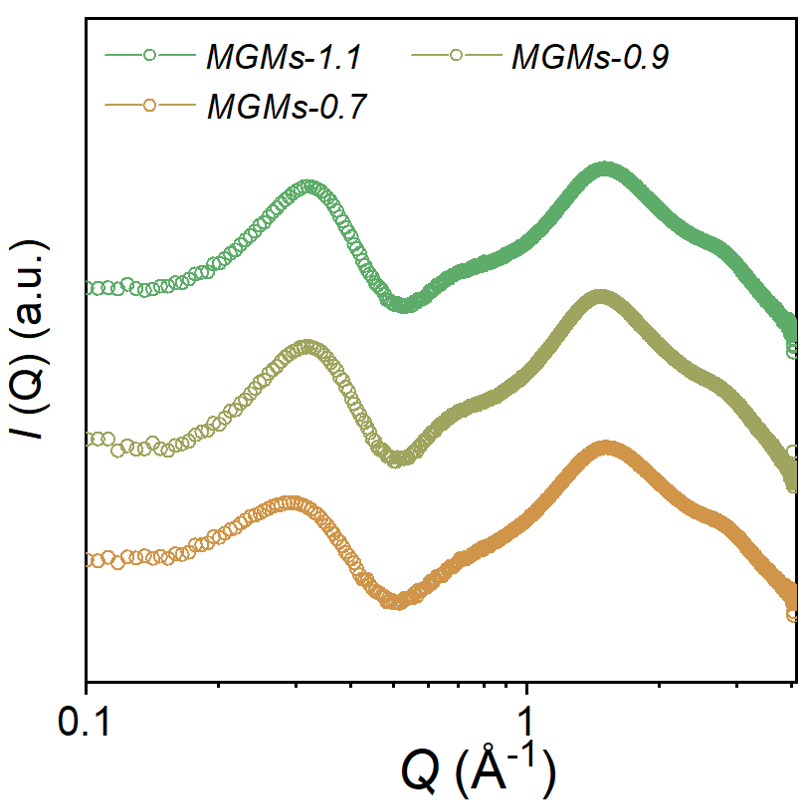


**Figure S10.** SAXS data of CPOSS-PEI composites with different loading of CPOSS.


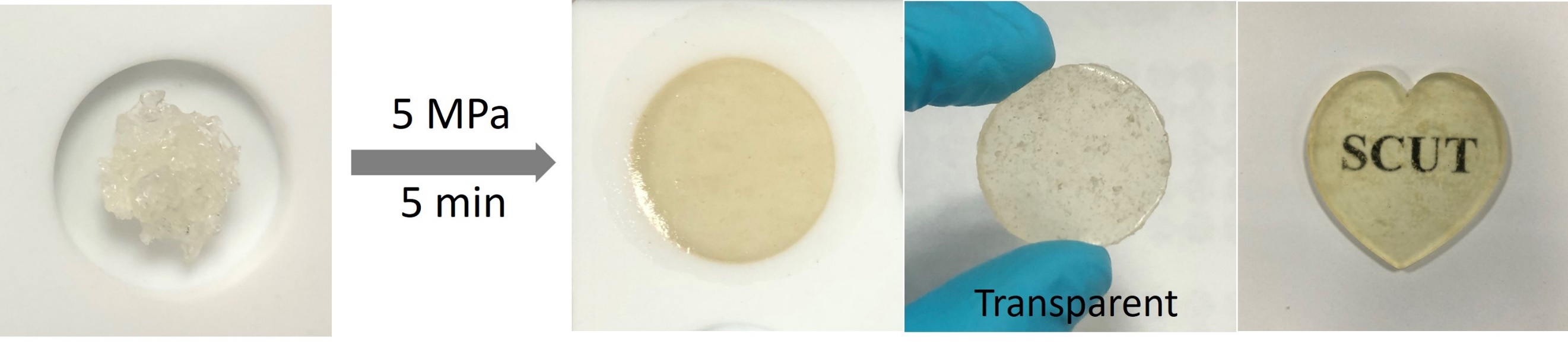


**Figure S11.** The processing of MGMs is performed under room temperature. The sample is processed in the designed mold under a pressure of 5 MPa for 5 min and can be feasibly processed into different shapes.


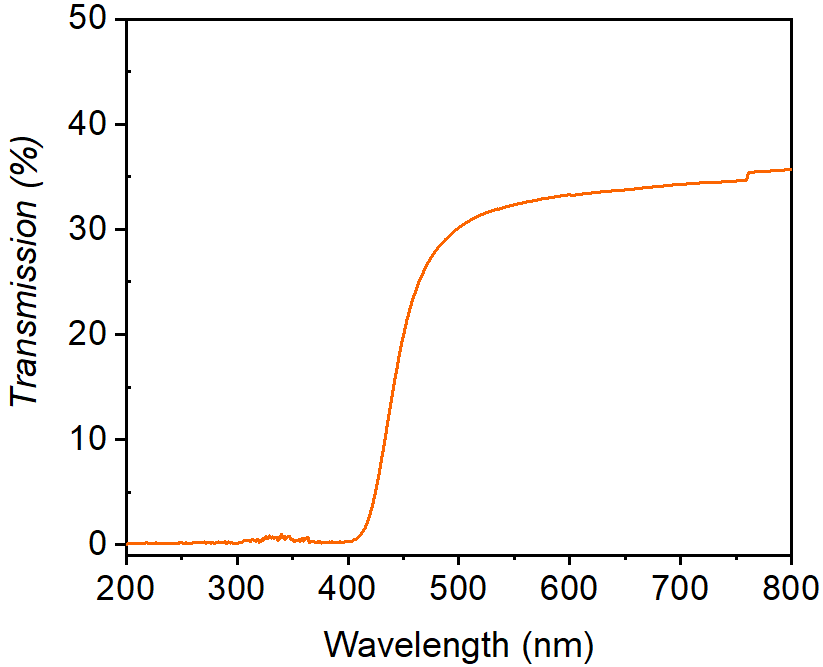


**Figure S12.** The transmittance curve of MGMs-1.0 film with a thickness of 0.5 mm.


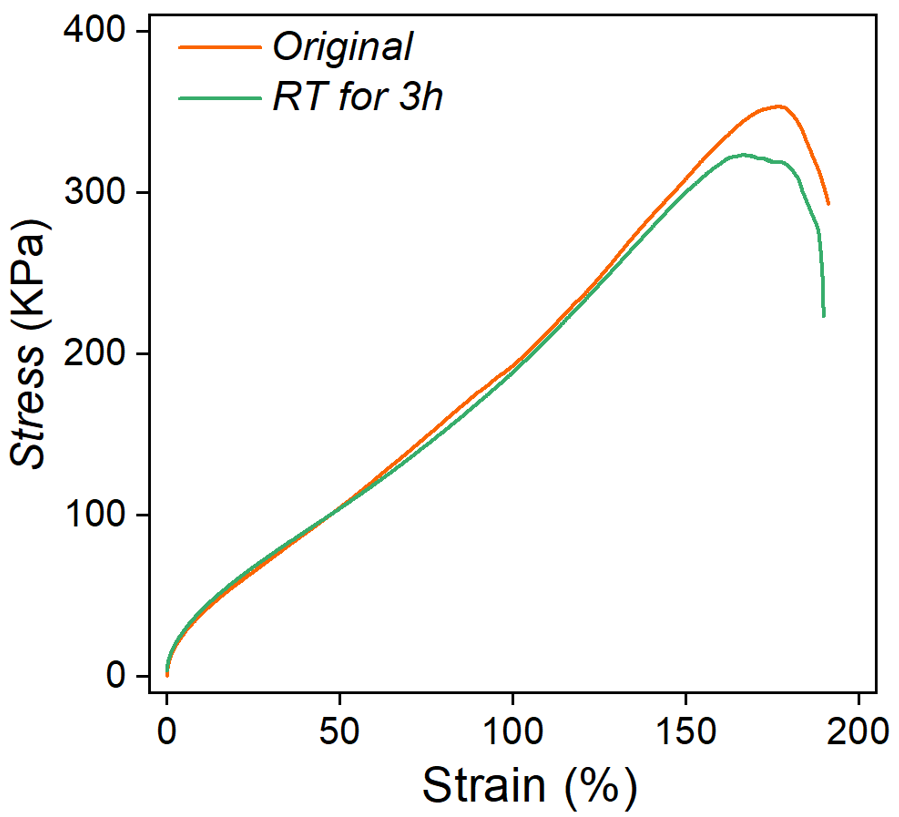


**Figure S13.** The strain-stress curves for cut sample after healing in comparison to the original MGMs sample.


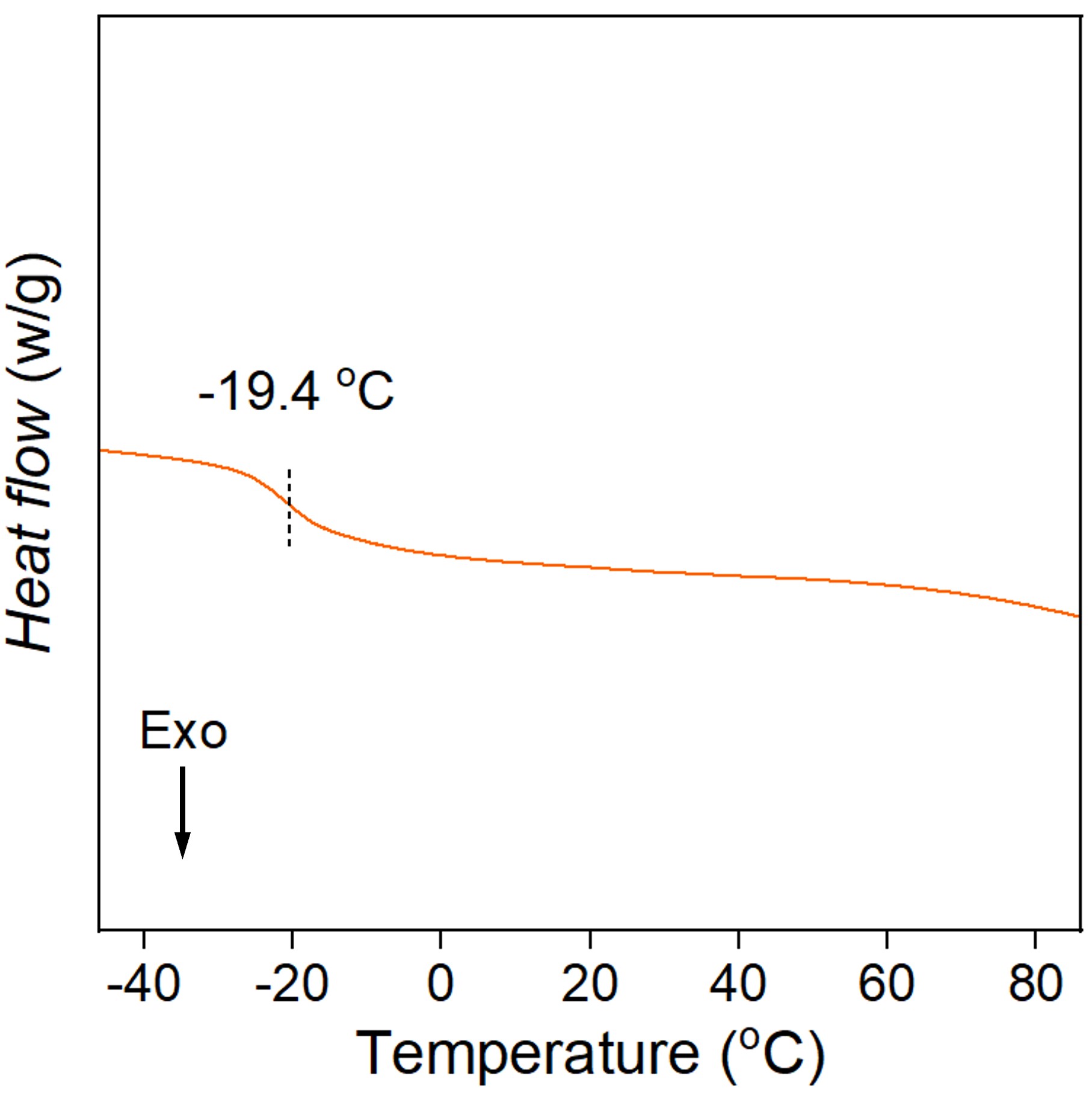


**Figure S14.** DSC curve of MGMs. The low *T_g_* further indicated the high mobility of the chain segments inside MGM, contributing to promising self-healing properties and processability of MGMs at room temperature.


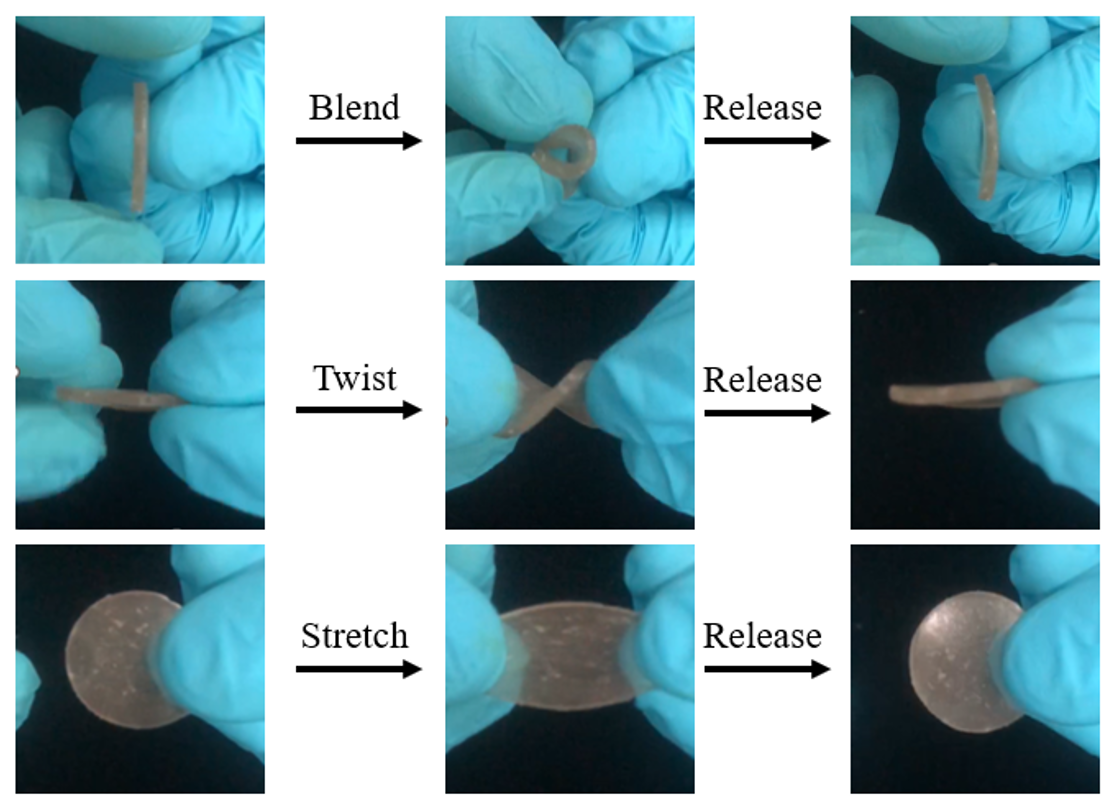


**Figure S15.** MGMs showed high elasticity under deformation such as bending, twisting, and stretching.


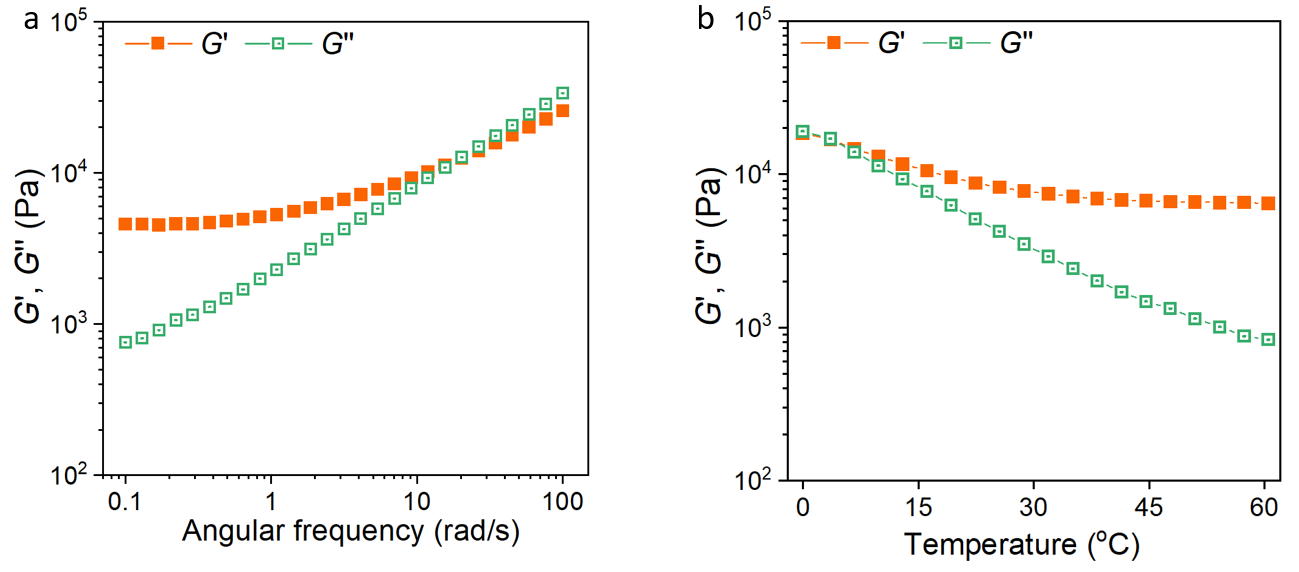


**Figure S16.** (a) Frequency sweep of MGMs with a shear strain of 0.1% at RT. (b) Temperature sweep of MGMs with an angular frequency of 1 rad s^-1^. Since the MGMs possesses some moisture that PEI keeps at room temperature, varying the temperature may cause the system structure to change and the time-temperature equivalence principle is no longer satisfied. Also, high temperature may cause PEI decomposition. Therefore, only the frequency scan at room temperature and the temperature scan test below 60 ℃ were performed.


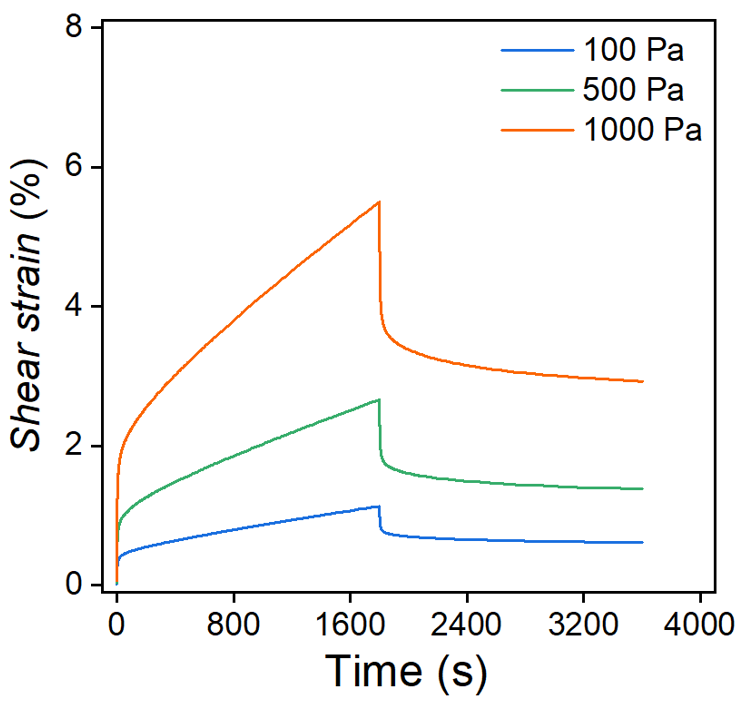


**Figure S17.** The creep curves of MGMs at different stress.


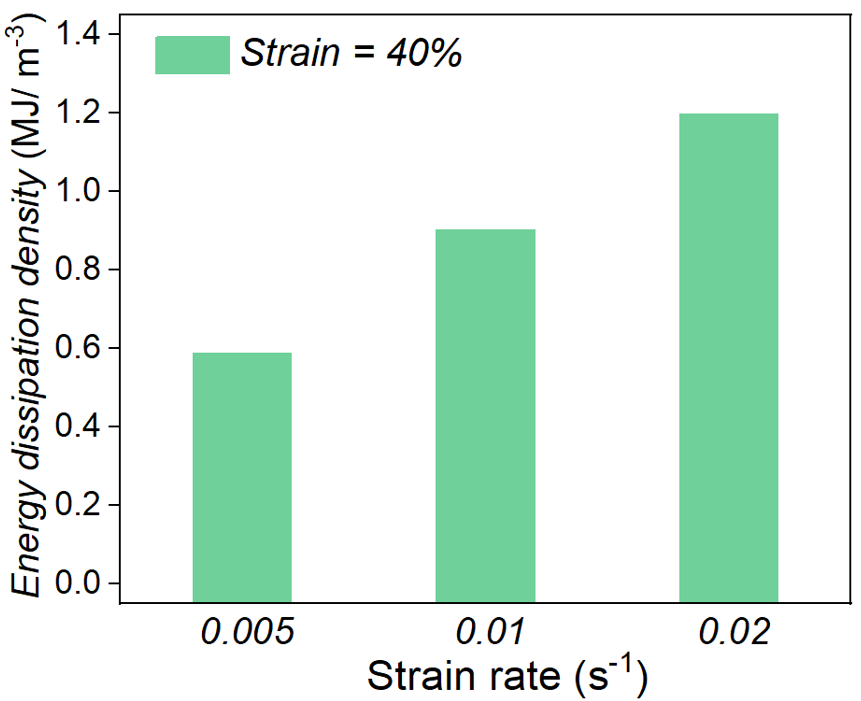


**Figure S18.** Rate-dependent energy dissipation density of MGMs.


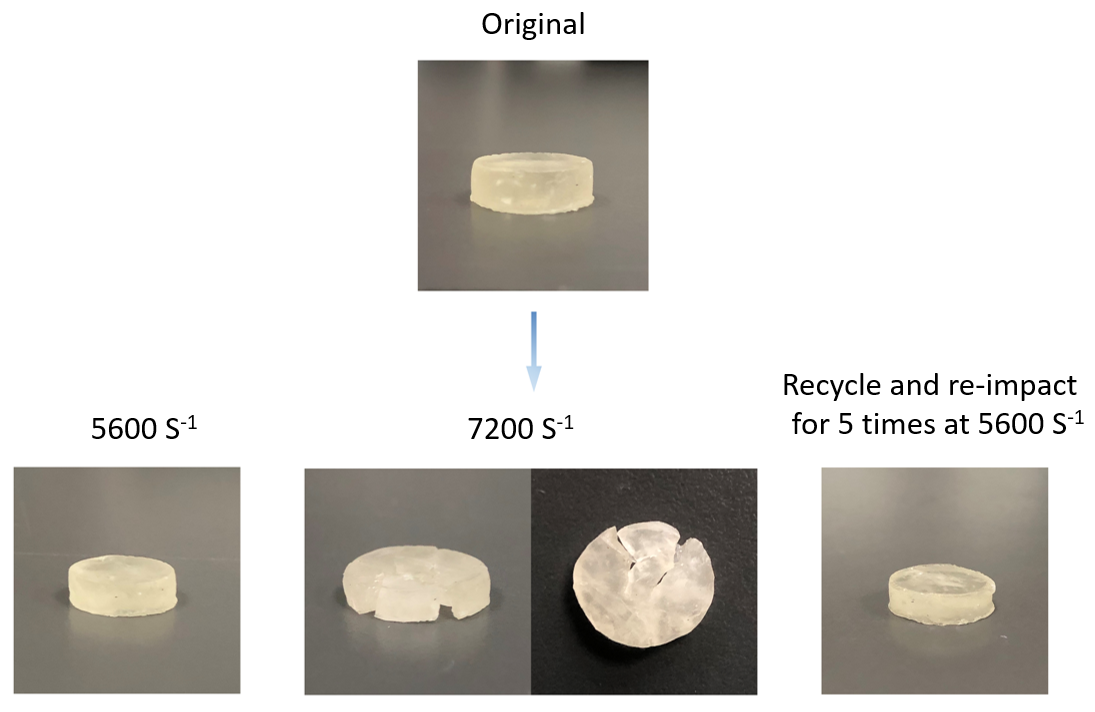


**Figure S19.** Digital photographs for the cylinder specimens and the failure patterns after dynamic Compression (SHPB) at different strain rates.


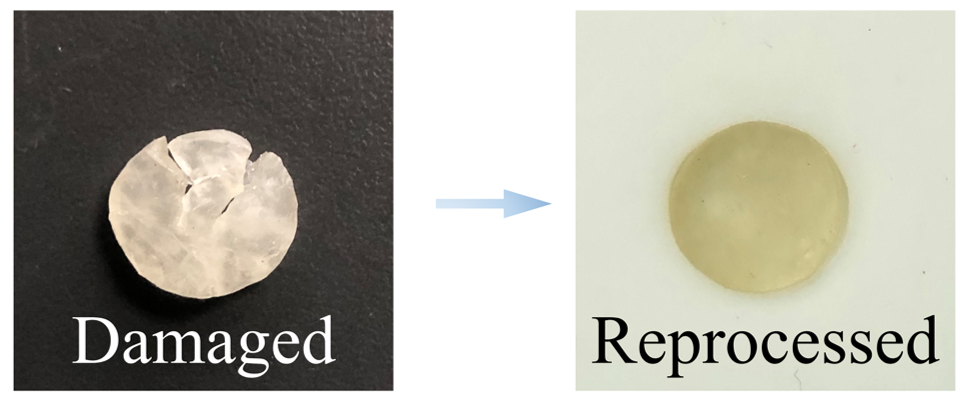


**Figure S20.** The damaged MGMs after SHPB tests was facilely reprocessed via pressure processing at 5 MPa, RT for 5 min.


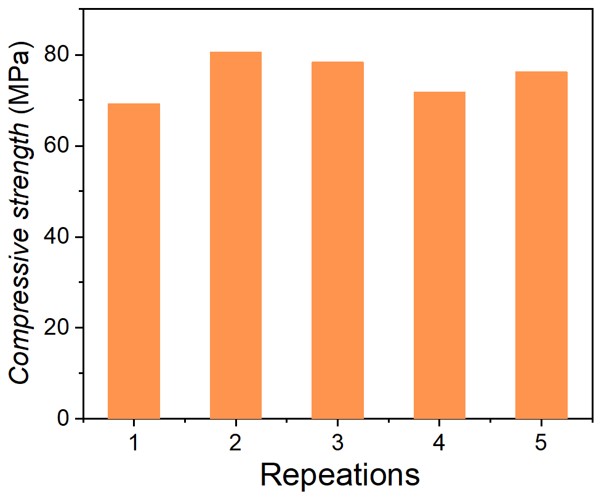


**Figure S21.** Compressive strength of MGMs under continuous impact at 5200 s^-1^.


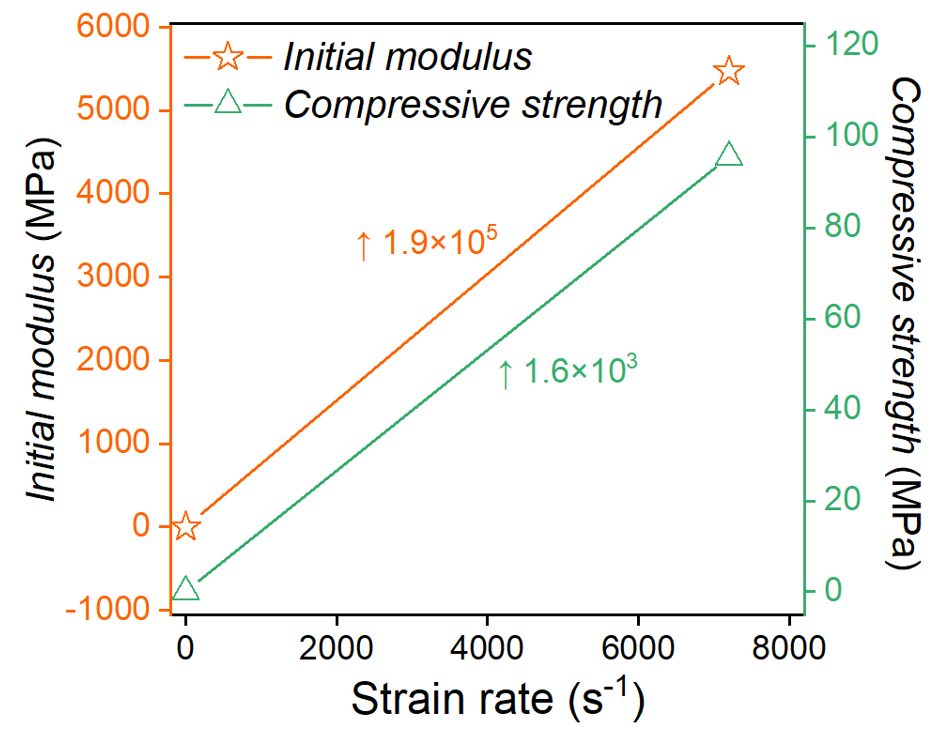


**Figure S22.** Dependence of initial modulus and compressive strength on strain rate of MGMs. Compared to low strain rate (0.005 s^-1^), the compressive strength and initial modulus of the material were increased by ca. 1.6×10^3^ and 1.9×10^5^ times at high strain rates (7200 s^-1^), respectively. The high modulus and strength of the under high-speed impact originated from strain hardening of high density crosslinking physical networks filled with hard CPOSS-aggregated nanodomains, providing enough modulus and strength for anti-resistance.


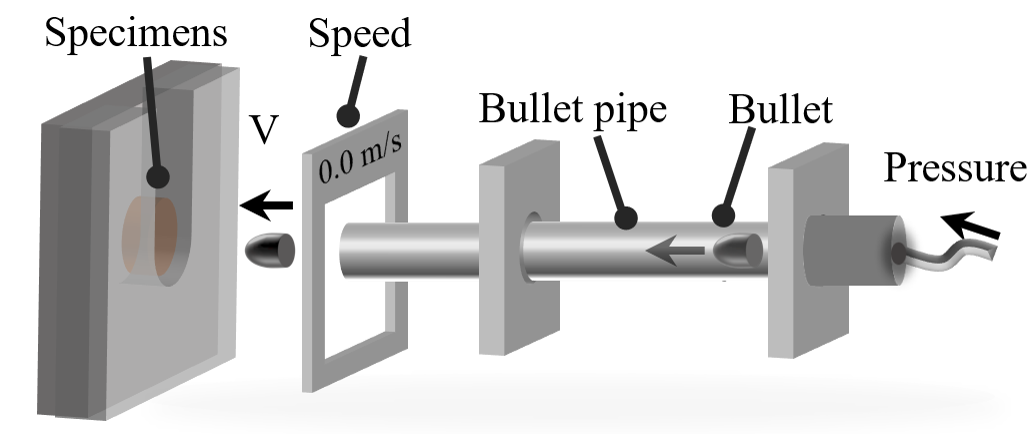


**Figure S23.** Schematic illustrating the air gun projectile system. It mainly includes bullet and speed measurement. The MGMs were disclike specimens with a thickness of 6.2 mm and a diameter of 30 mm.


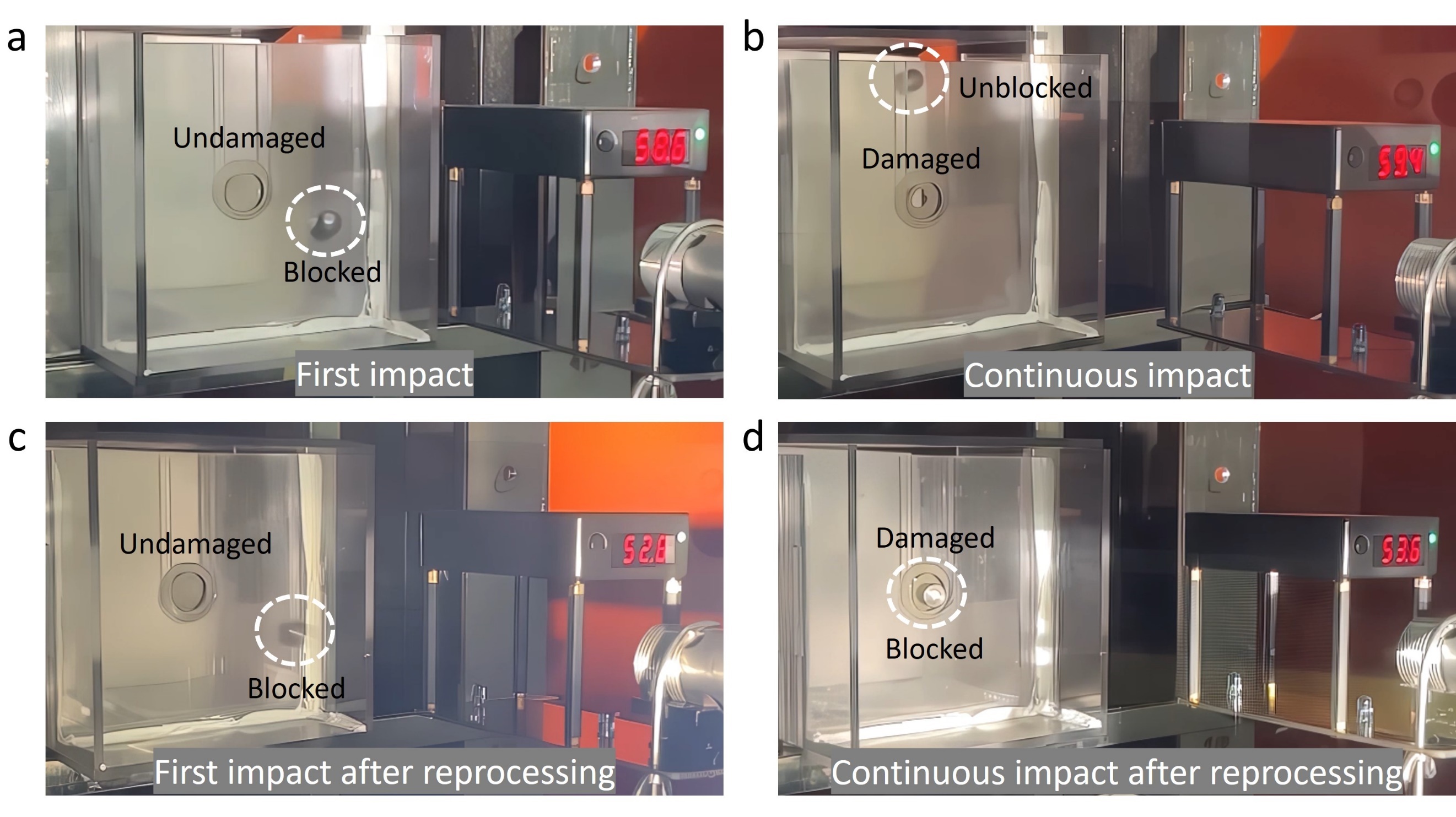


**Figure S24. Snapshots of bullets impacting POSS at different speeds.** (a) The first impact on MGMs (58.6 m s^-1^). (b) The further impact on MGMs (59.4 m s^-1^). (c) The first impact on reprocessed MGMs (52.6 m s^-1^). (d) The further impact on reprocessed MGMs (53.6 m s^-1^). Steel projectile with speeds of 58.6 m s^-1^ can be effectively blocked by the MGMs film (**Figure S24a** **and Movie S1**). It takes a further impact at 59.4 m s^-1^ to cause damage to MGMs, revealing the superb anti-impact capacity of MGMs (**Figure S24b** **and Movie S2**). The reprocessed MGMs can still effectively resist the continuous impact of 52.6 m s^-1^ (**Figure S24c** **and Movie S3**) and 53.6 m s^-1^ (**Figure S24d** **and Movie S4**), exhibiting super-fast recoverability and superior impact resistant ability.


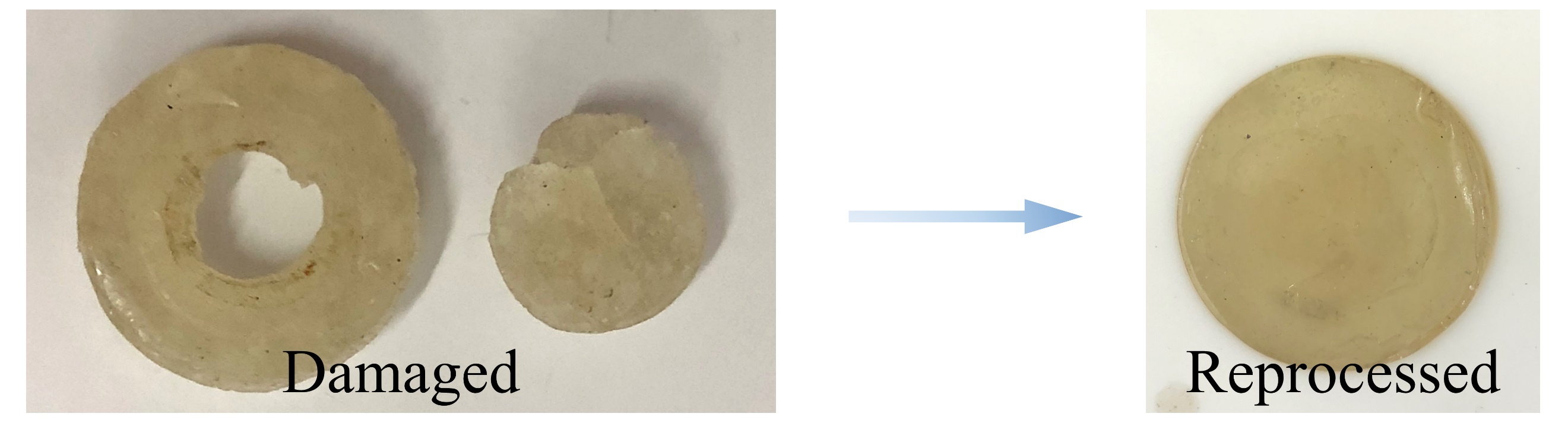


**Figure S25.** The damaged MGMs after air gun projectile tests was simply and quickly reprocessed in the mold at RT for 5 min under the pressure of 5 MPa.


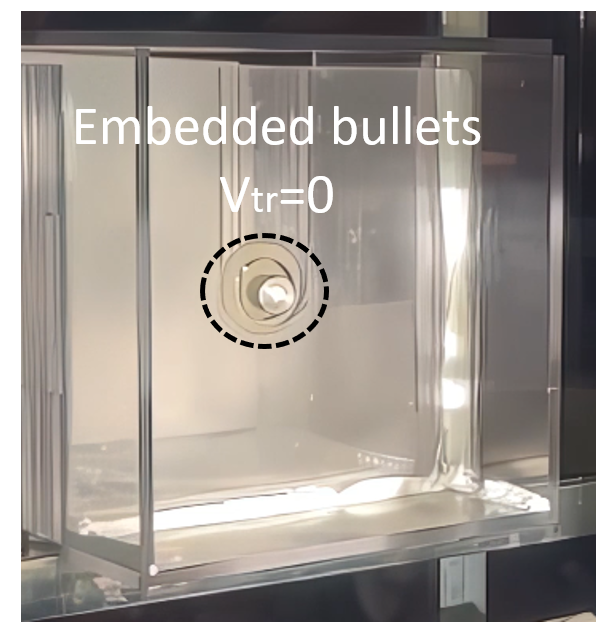


**Figure S26.** The calculation of effective mass and volume of specimens dissipating impact energy. The bullet of 53.6 m s^-1^ was blocked by the MGMs and embedded in the sample. The transmission velocity (*V_tr_*) of the bullet can be considered as 0. The diameter of the effective part of the specimen dissipating energy is approximately equal to the diameter (*d* = 12.4 mm) of the bullet. Therefore, effective mass and volume of specimens were calculated as 1.06 g and 0.75 cm^3^, respectively.

**Determination of the impact strength for MGMs**

**(1) Impact strength from SHPB tests**

Based on split-Hopkinson pressure bar (SHPB) experiments, we had access to the impact strength of our materials. *V*_p_ was the total volume and *S*_p_ was the bottom area of the disc-like Tri-POSS sample. The energy was absorbed and dissipated by the whole sample. MGMs samples were disc-like specimens with a thickness of 4 mm (d) and a diameter of 10 mm (2R).

$$V_{p}=S_{P}d$$

The work done by split-Hopkinson pressure bar during the impact is calculated using the following formula:

$$W_{SHPB}=Fx=\left( \sigma S_{P} \right)\cdot\left( d\varepsilon\right)=\left( \sigma\varepsilon\right)\cdot\left( S_{P}d \right)$$

Where σ and ε were the corresponding stress and strain respectively. The absorbed energy per unit volume of specimen (W_v_) was calculated as

$$W_{V}=\frac{W_{SHPB}}{V_{P}}=\frac{\left( \sigma\varepsilon\right)\cdot\left( S_{P}d \right)}{S_{P}d}=\sigma\varepsilon=\int{f\left( \varepsilon\right)}_{Stress-strain}$$

$$\text{where}\int{f\left( \varepsilon\right)}_{Stress-strain}\text{ }\text{represented the }\text{integral area}\text{ of stress-strain curve}$$

Subsequently, the absorbed energy per unit volume of specimen (W_m_) was calculated as

$$W_{m}=\frac{W_{V}}{\rho}$$

Where *ρ* = 1.42 g cm^-3^ represents the density of the sample. The calculated results were summarized in **Table S1**.

**Table S1.** Data for the split-Hopkinson pressure bar (SHPB) experiments.

| Strain rate  (s^-1^) | Integral area  (m/s) | W_m_  (J/g) | W_v_  (J/cm^3^) |
| --- | --- | --- | --- |
| 1700 | 2.54 | 1.79 | 2.54 |
| 2700 | 10.68 | 7.52 | 10.68 |
| 3500 | 17.66 | 12.44 | 17.66 |
| 4500 | 21.72 | 15.30 | 21.72 |
| 5600 | 32.85 | 23.13 | 32.85 |
| 7200 | 42.55 | 29.96 | 42.55 |

**(2) Impact strength from air gun projectile impact study**

The impact strength provides quantitative information for MGM’s impact-resistant capacity. Details about the calculation of impact strength from air gun projectile impact were summarized as below. The initial velocity ($V_{in}$) can be readable from the velocity detector. Since the projectile was entirely blocked by our MGM film, the terminal velocity (*V_t_*) was reasoned to be 0 m/s. Therefore, the kinetic energy loss of the projectile during the impact process was therefore quantified as:

$$\Delta E_{k}=E_{k2}-E_{k1}=\frac{m_{b}\left( V_{in}^{2}-0^{2} \right)}{2}$$

the impact strength (*G_im_*) can be defined as the kinetic energy dissipation for unit mass or volume:

$$\begin{aligned} &G_{im}^{a}=\frac{\Delta E_{k}}{m_{p}}=\frac{m_{b}\left( V_{in}^{2}-0^{2} \right)}{2m_{p}} \\ &\text{ or }G_{im}^{b}=\frac{\Delta E_{k}}{V_{p}}=\frac{m_{b}\left( V_{in}^{2}-0^{2} \right)}{2V_{p}} \end{aligned}$$

where $m_{p}$ and $V_{p}$ represent the mass and volume of the MGM specimen.

In the first case, we hypothesize the kinetic energy was absorbed and dissipated by the whole sample. $m_{p}$ and $V_{p}$ represent the total mass and volume of the MGMs sample, respectively. Therefore, the value of $G_{im}^{a}$ and $G_{im}^{b}$ can be afforded, which is listed below in **Table S2.**

In the second case, we hypothesize the input energy was totally dissipated by the failure region inside the samples. Herein, $m_{p}$ and $V_{p}$ represented the effective mass and effective volume of the specimens, respectively, which were calculated as 1.06 g and 0.75 cm^3^ from the failure patterns in **Figure S21**. The calculated results were summarized in **Table S2**.

**Table S2.** Data for the air gun projectile impact study.

| *V_in_*  (m s^-1^) | *V_t_*  (m s^-1^) | *ΔE_k_*  (J) | *G_im_*^a^  (J g^-1^) | *G_im_*^b^  (J cm^-3^) | *G_im_*^aa^  (J g^-1^) | *G_im_*^bb^  (J cm^-3^) |
| --- | --- | --- | --- | --- | --- | --- |
| 53.6 | 0 | 17.53 | 2.83 | 4.02 | 16.54 | 23.48 |

*G_im_^a^* and *G_im_^b^* represent the energy dissipation of the whole sample, while the *G_im_^aa^* and *G_im_^bb^* represent the energy dissipation of the effective part of the sample.


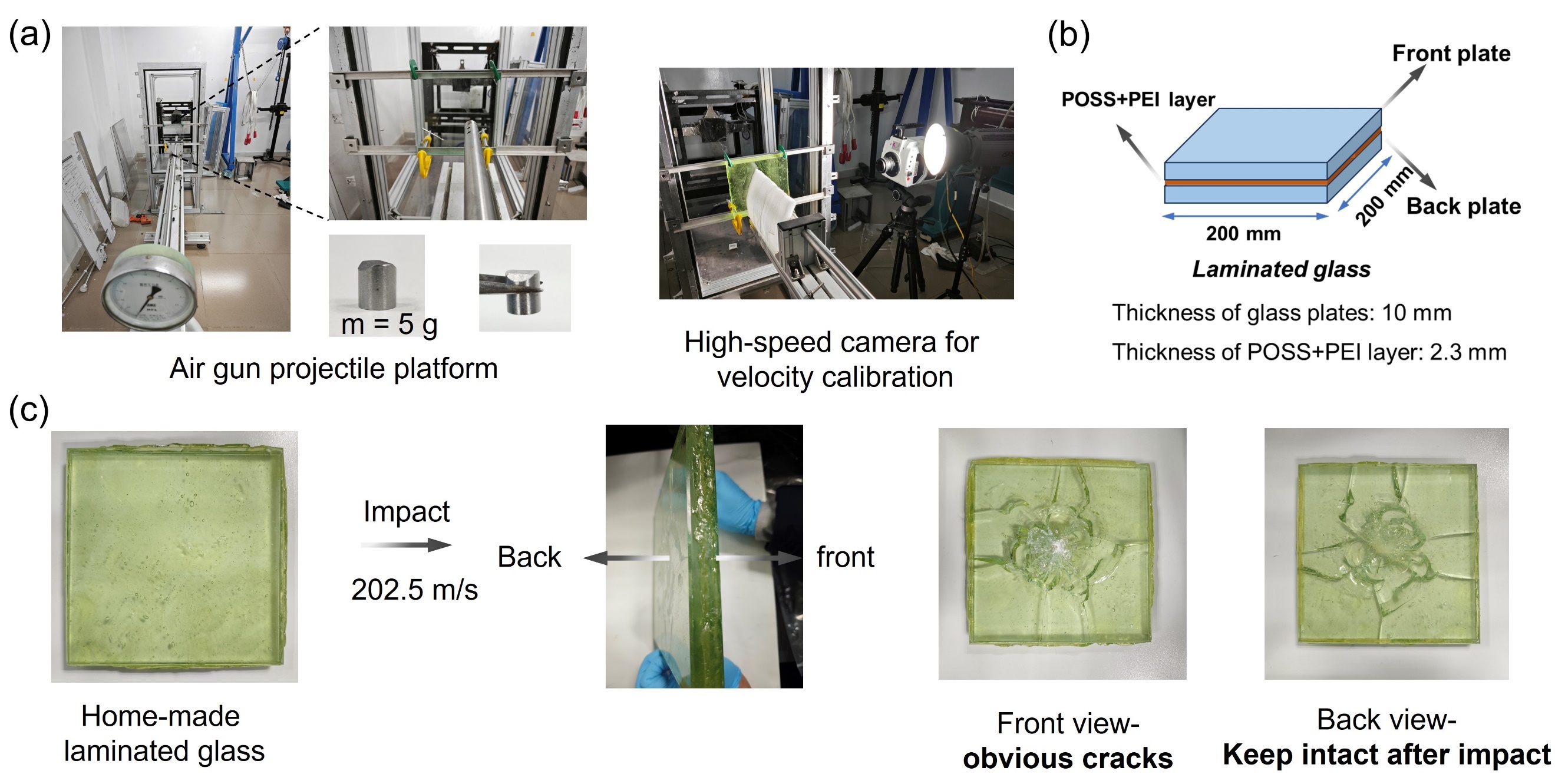


**Figure S27.** (a) Air gun projectile platform equipped with a high-speed camera is used for impact test. High-speed camera is exploited here for the calibration of the velocity of steel projectile. (b) Detailed structures of laminated glass specimen. The thicknesses of POSS/PEI layer and glass plate are 2.3 mm and 10 mm, respectively. (c) Left is the front view of the laminated glass before impact. For the impact test, the compressed air (0.9 MPa) is completely released at a critical time and the projectile is therefore abruptly accelerated. The velocity of projectile is determined to be 202.5 m s^-1^, calibrated by the high-speed camera. Pictures on the right show the laminated glass after impact. The back glass plate keeps intact after impacts, demonstrating the exceptional energy dissipation ability of our POSS/PEI layers.


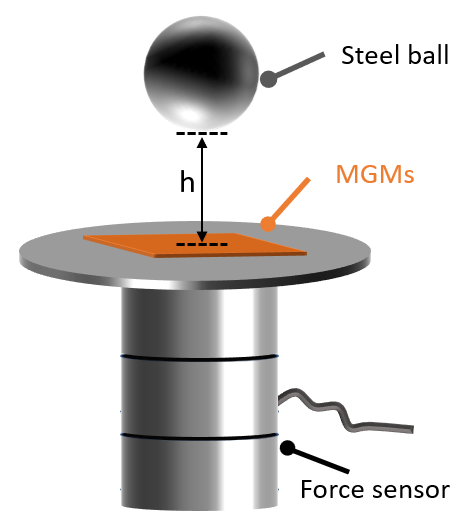


**Figure S28.** Schematic illustrating falling ball impact test system.


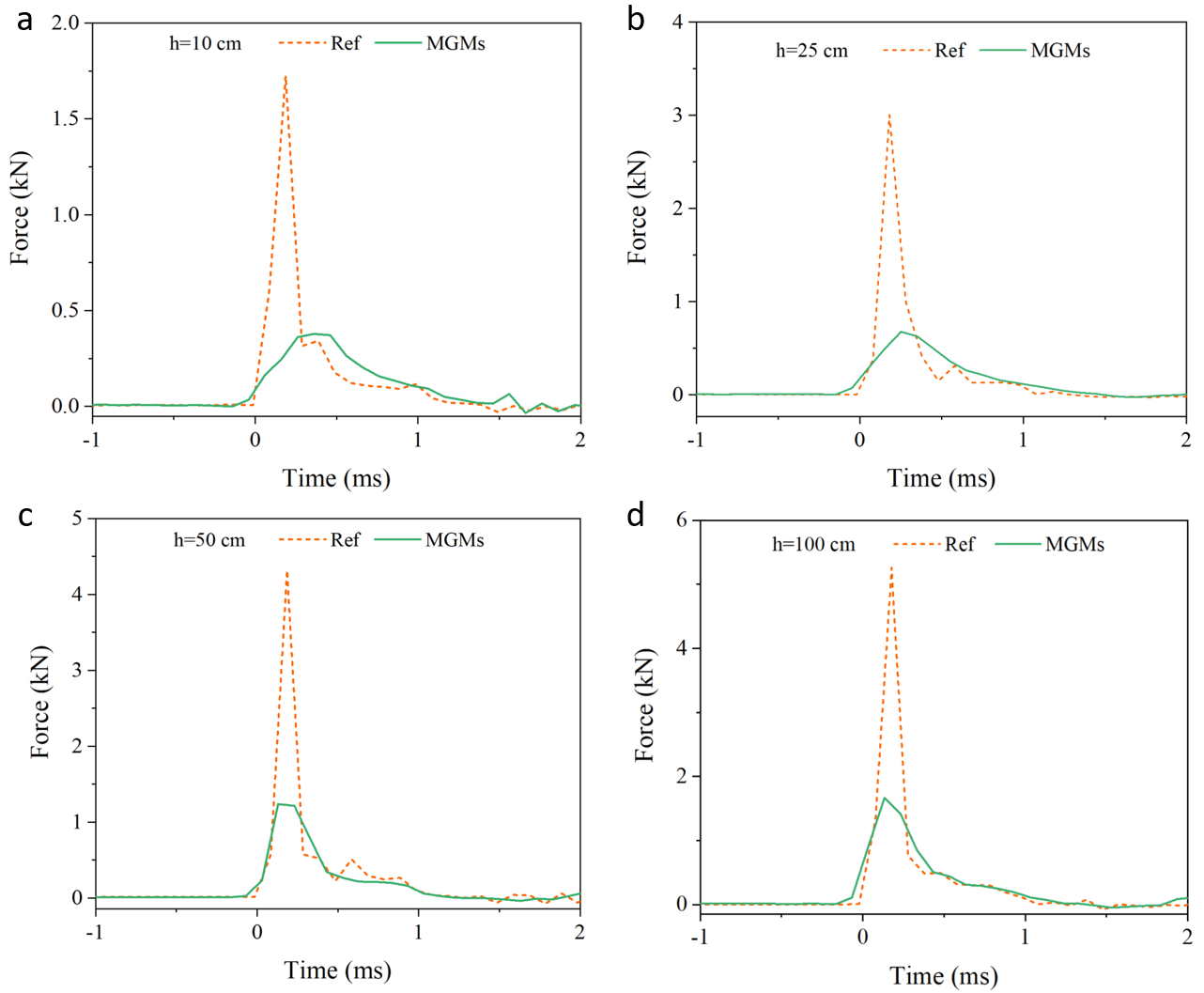


**Figure S29. Force-time curves of MGMs during impact process from different heights.** (a) 10 cm. (b) 25 cm. (c) 50 cm. (d) 100 cm. Note that the force signals of reference condition (Ref) represent direct impact of ball toward force sensor.


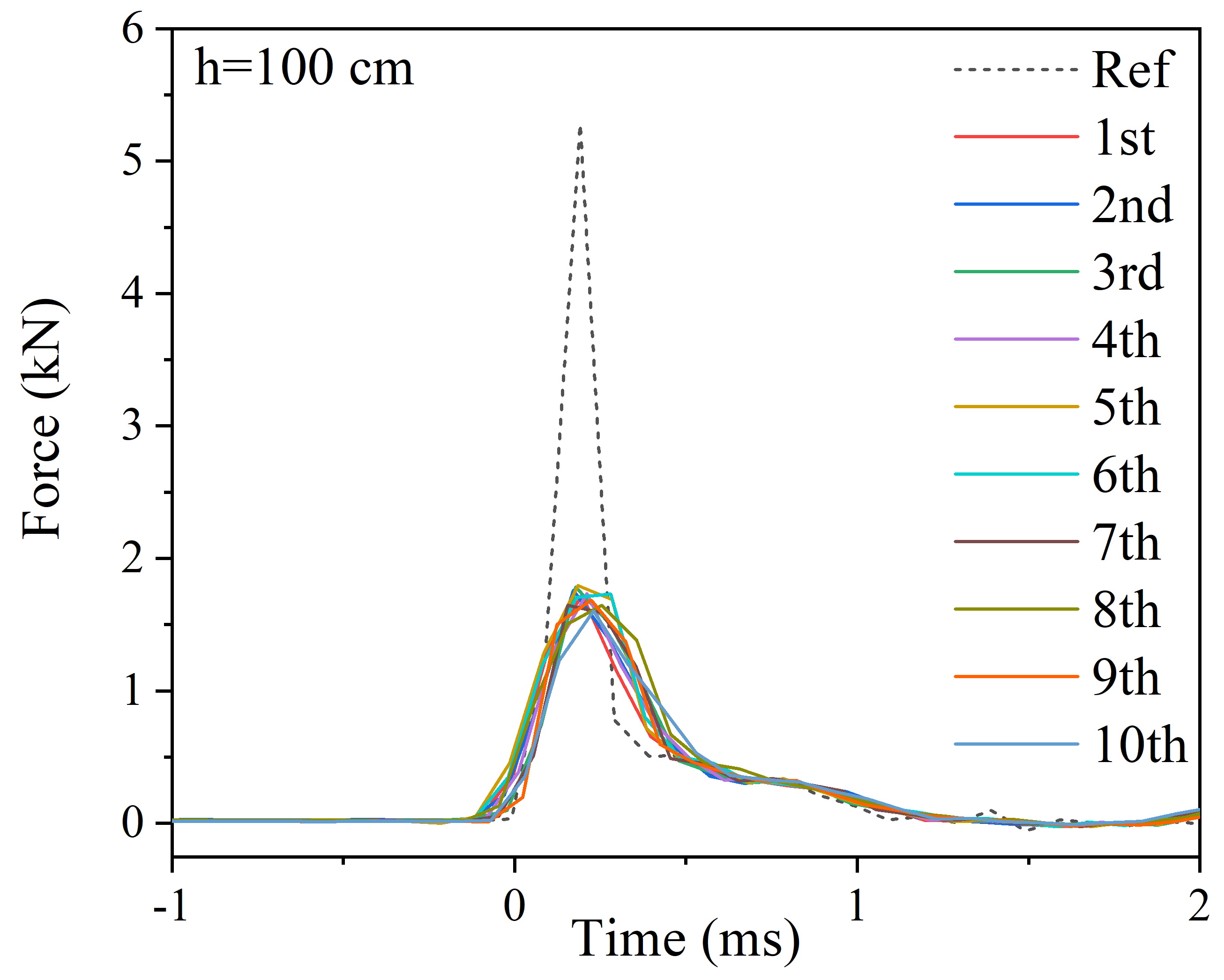


**Figure S30.** Force-time curves of MGMs during 10 repeated impact process from 100 cm.


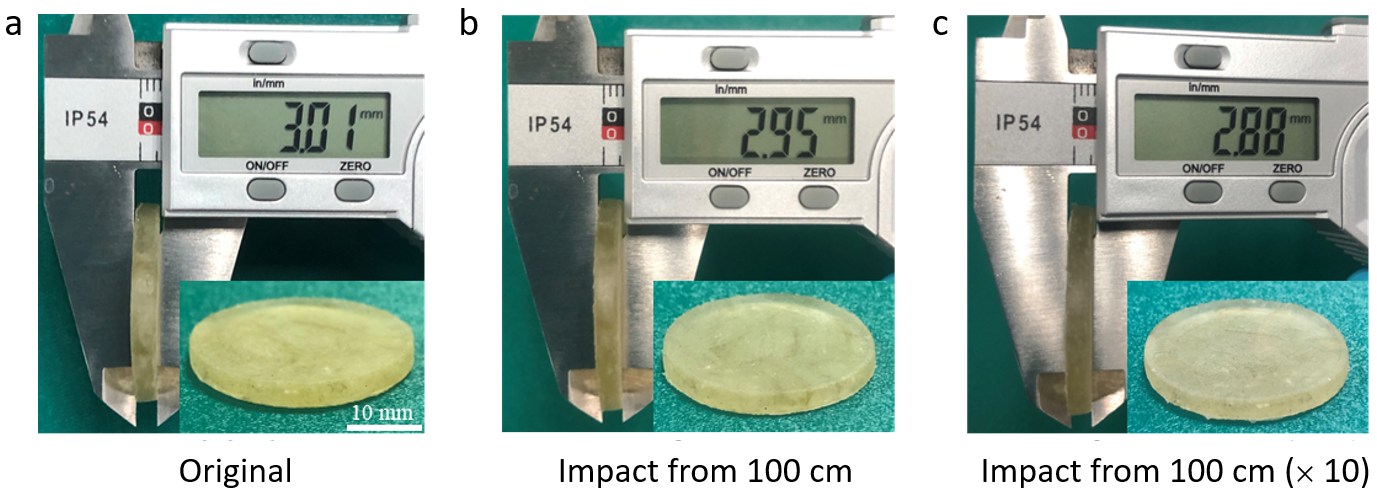


**Figure S31.** Surface morphologies and thickness of MGMs samples before and after impact. Scale bars, 10 mm. The photographs of MGMs after impact from 100 cm and repeated 100 cm remained an intact appearance and no noticeable shape change owing to the recoverable elastic deformation and excellent energy dissipation of MGMs, demonstrating excellent impact resistant ability and promising long-term reusability.


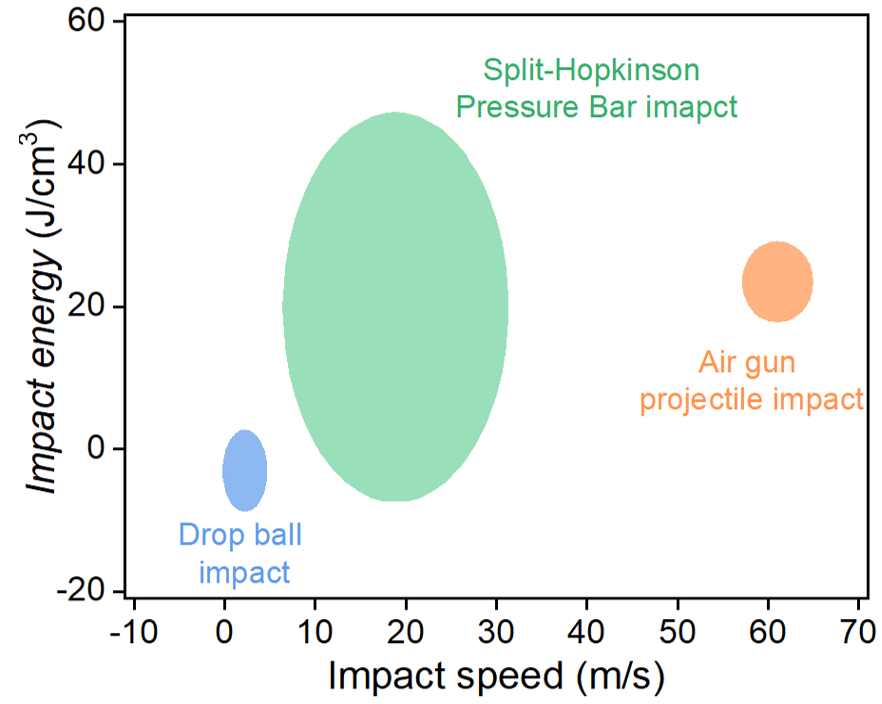


**Figure S32.** The impact energy on MGMs at different speed from various impact method. The multiple impact testing methods demonstrate that MGMs can withstand a wide range of energy and velocity impacts, showing promise for diverse application circumstances.


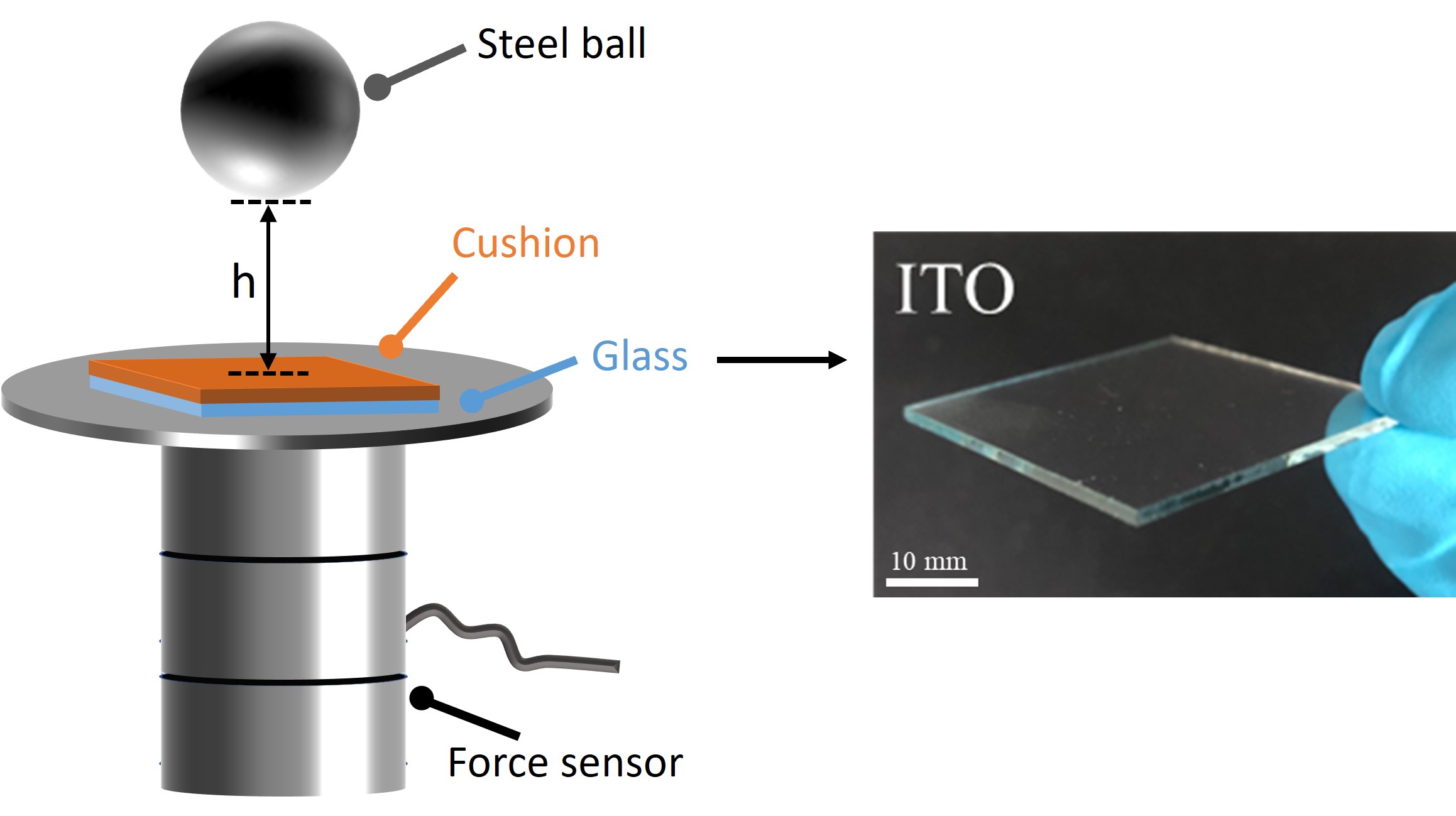


**Figure S33.** Schematic illustrating the falling ball impact protection test system and the picture of Indium-Tin-Oxide (ITO) glass (dimension: 30 mm×30 mm×1mm). Scale bars, 10 mm.


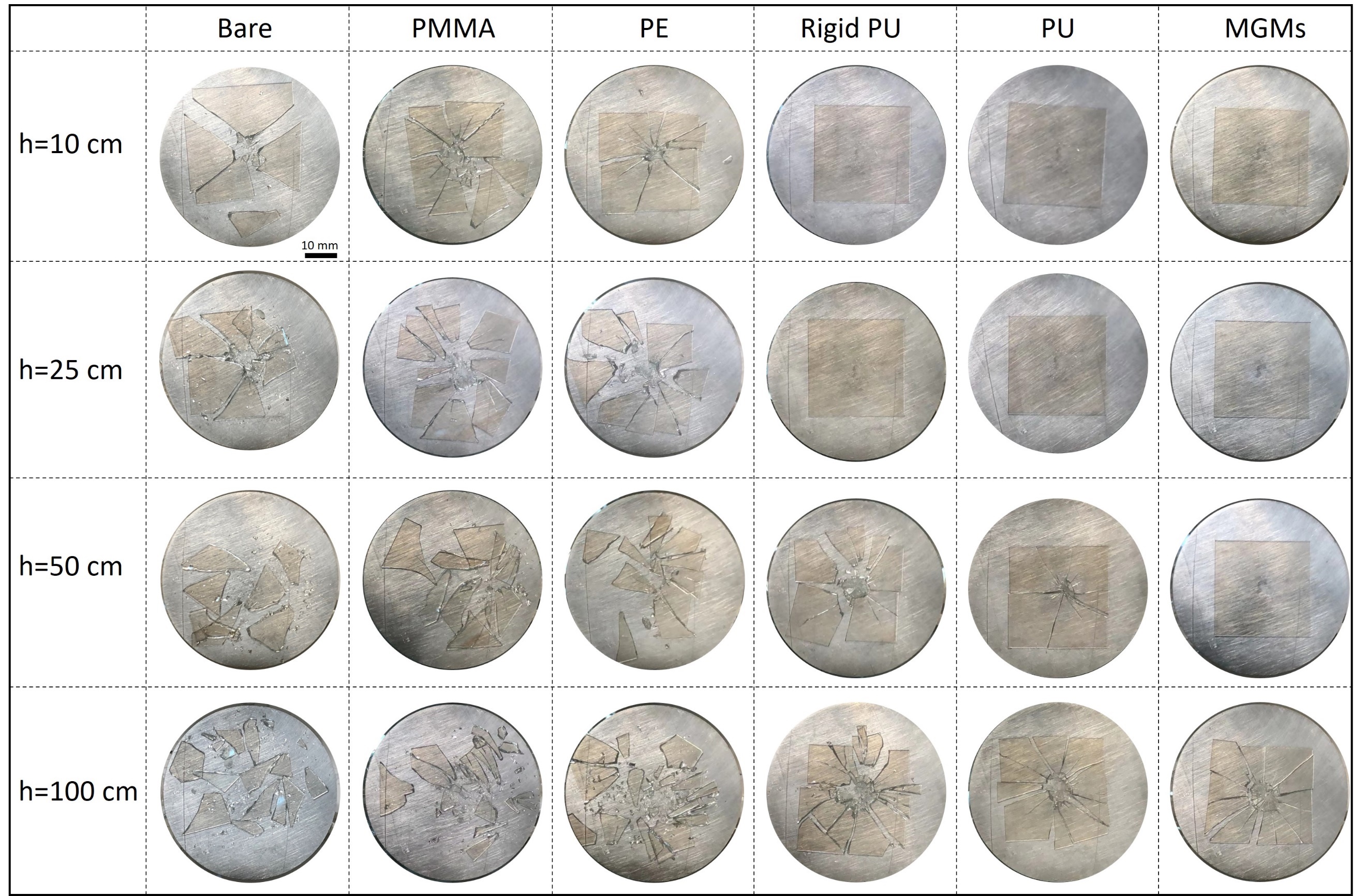


**Figure S34.** Morphologies of impacted glass protected with nothing, PMMAPE, PE, rigid PU, PU and MGMs from different falling height. Scale bars, 10 mm. Under the impact of 50 cm, the glass protected by MGMs remained intact, while the glass protected by other polymeric materials were broken. Under the impact at 100cm, the high impact energy causes all the glass to break.


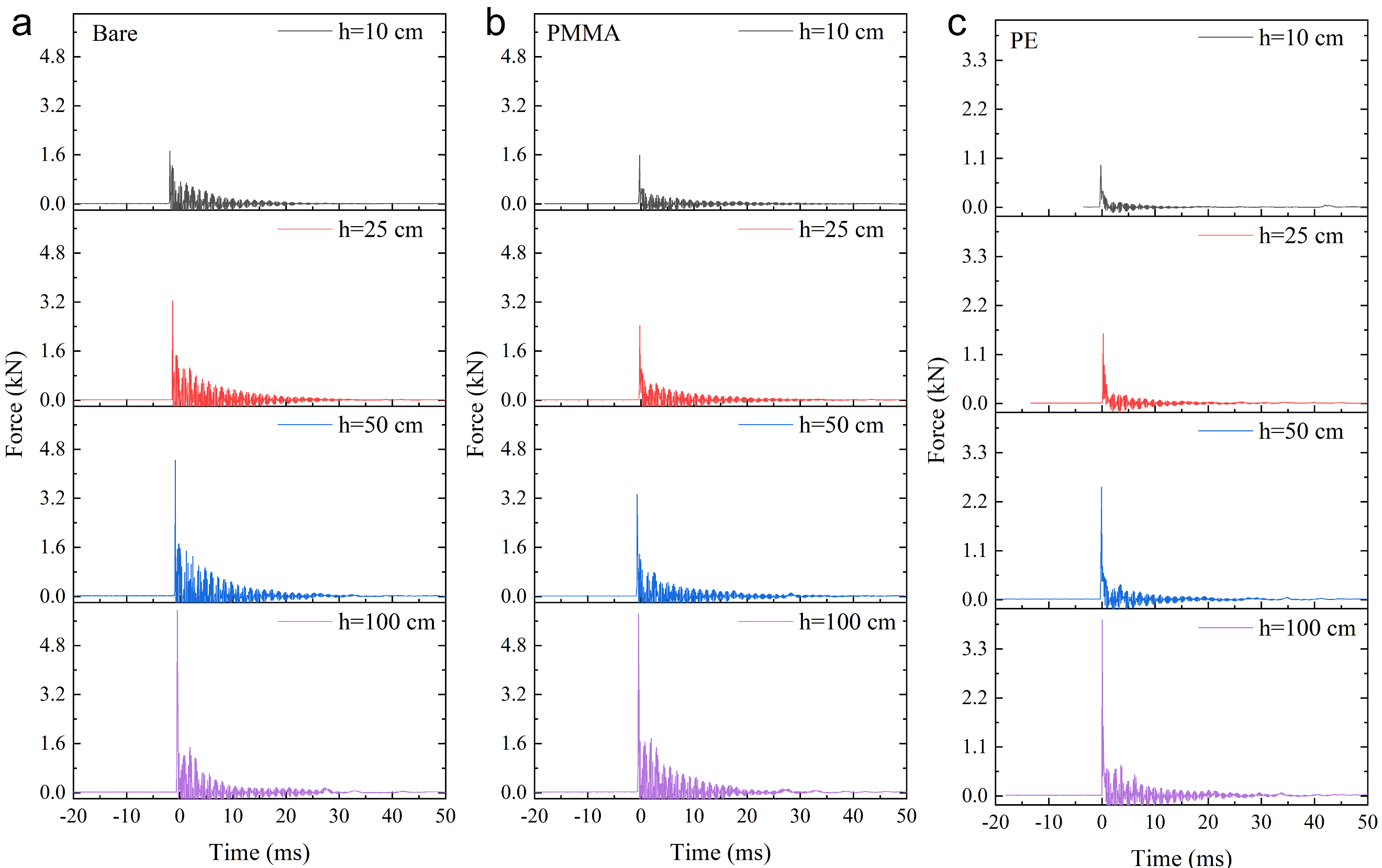


**Figure S35.** Force-time curves of impacted glass protected with (a) nothing, (b) PMMA and (c) PE from different falling height.


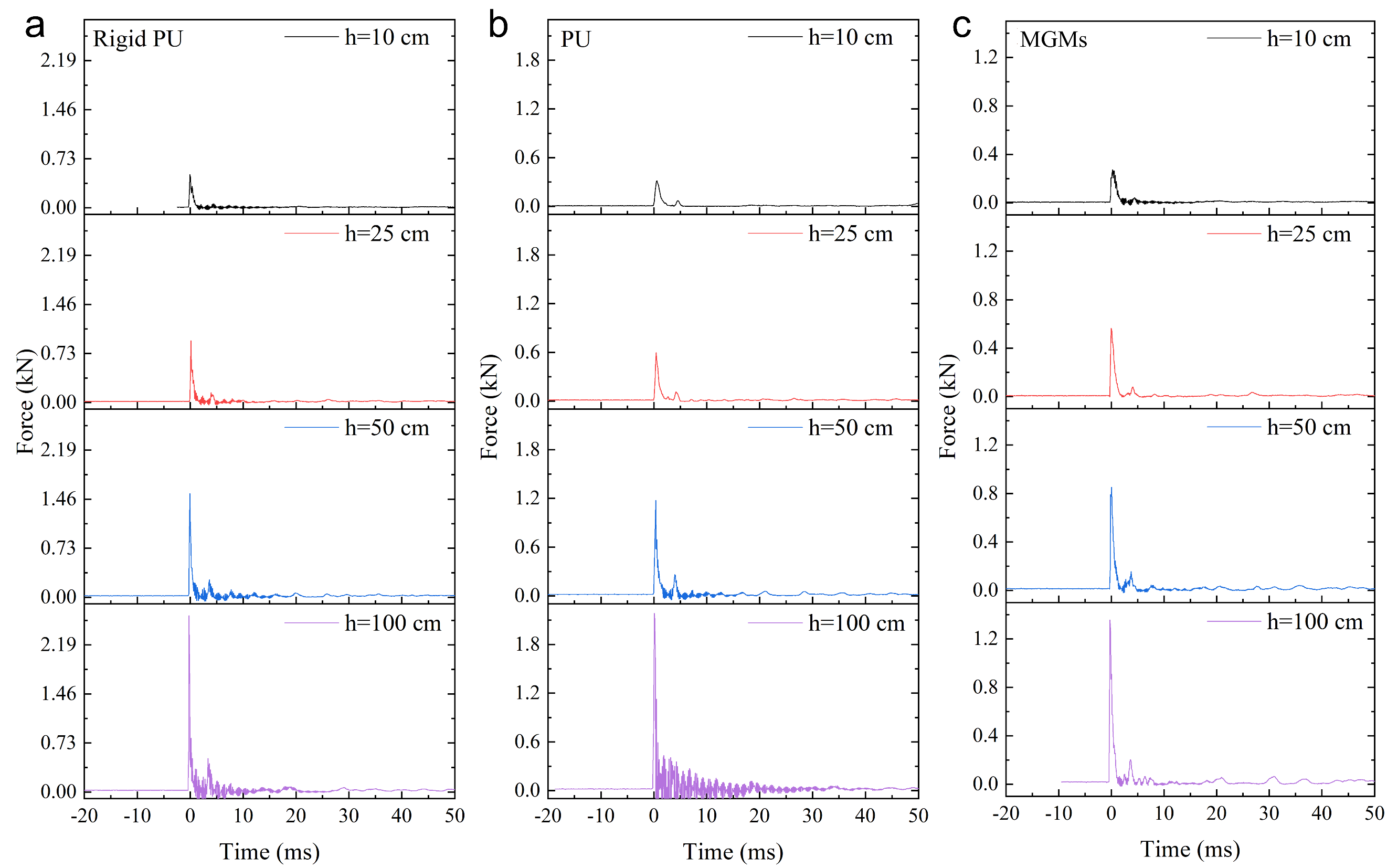


**Figure S36.** Force-time curves of impacted glass protected with (a) rigid PU, (b) PU and (c) MGMs from different falling height.


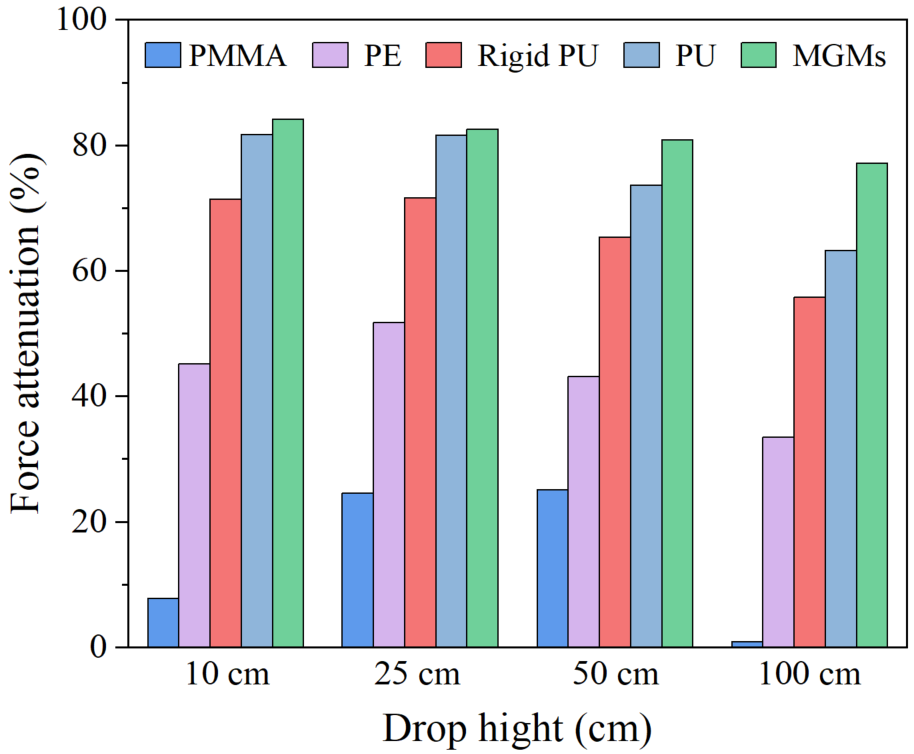


**Figure S37.** The force attenuation of various cushioning materials from different falling heights. MGMs shows higher force attenuation coefficient compared to other polymeric materials. As the impact height increases, MGMs can maintain a high force attenuation coefficient of 80%, demonstrating robust impact resistance.


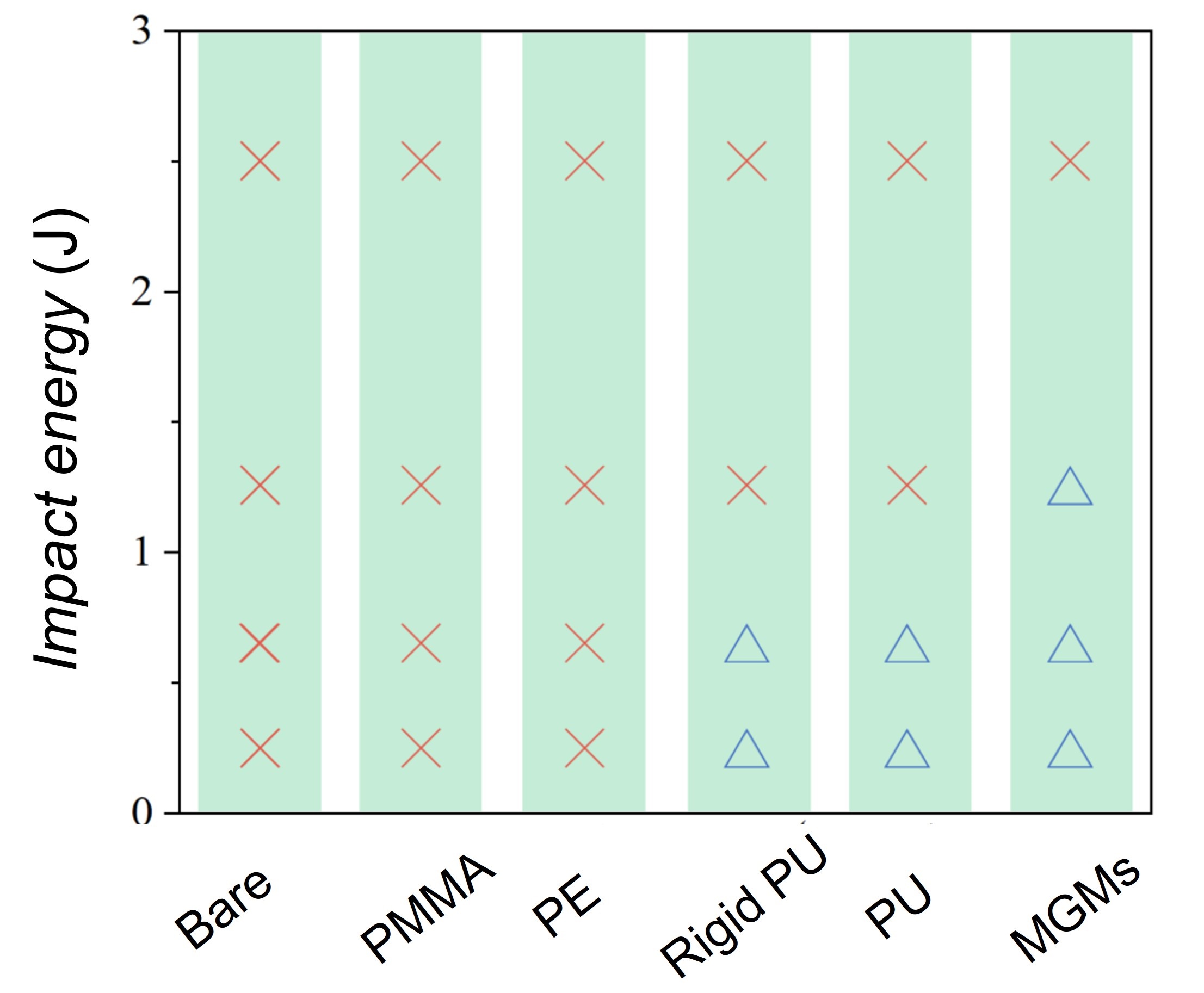


**Figure S38.** Relationships between the applied *E*_I_ and impact resistance of impacted ITO glass protected with nothing, PMMAPE, rigid PU, PU and POSS _1.0_ from different falling height. Open blue triangles represent *E*_I_ applied when the glass keeps intact after impact. Red crosses represent *E*_I_ applied when the ITO glass beaks after impact.


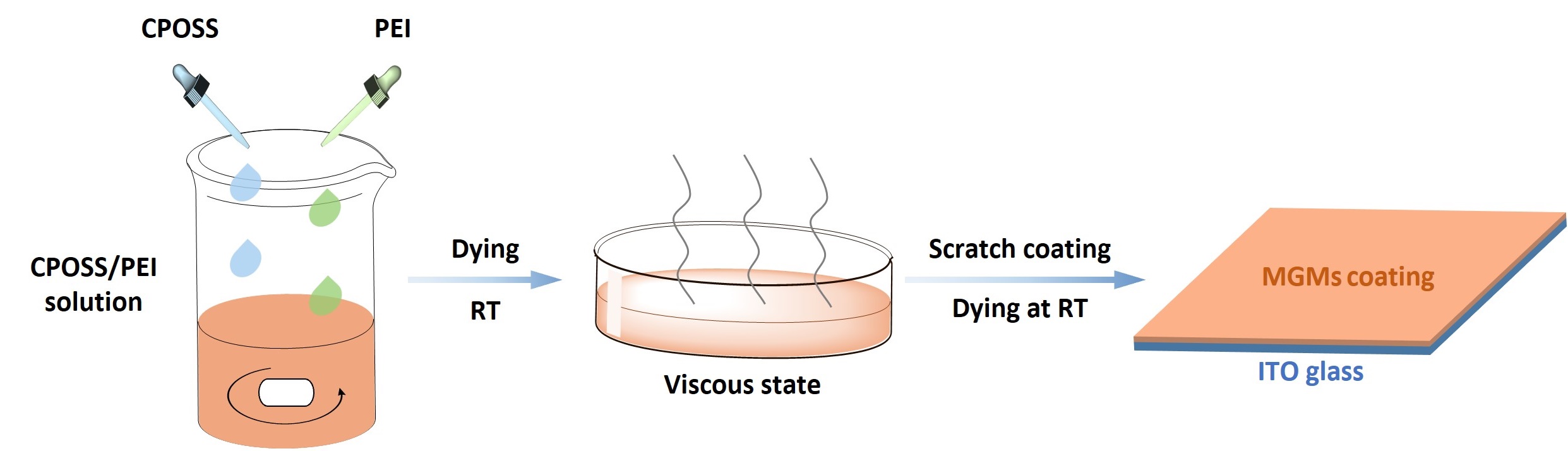


**Figure S39.** Preparation process of MGMs coating on ITO glass. The CPOSS and PEI solutions were mixed to obtain the CPOSS/PEI mixture, and the solvent was evaporated at room temperature for ~24 h to obtain the viscous CPOSS/PEI composites. Viscous composites were scraped onto 1 mm glass and dried at room temperature for ~48 h to form a uniform coating of approximately 0.7 mm.


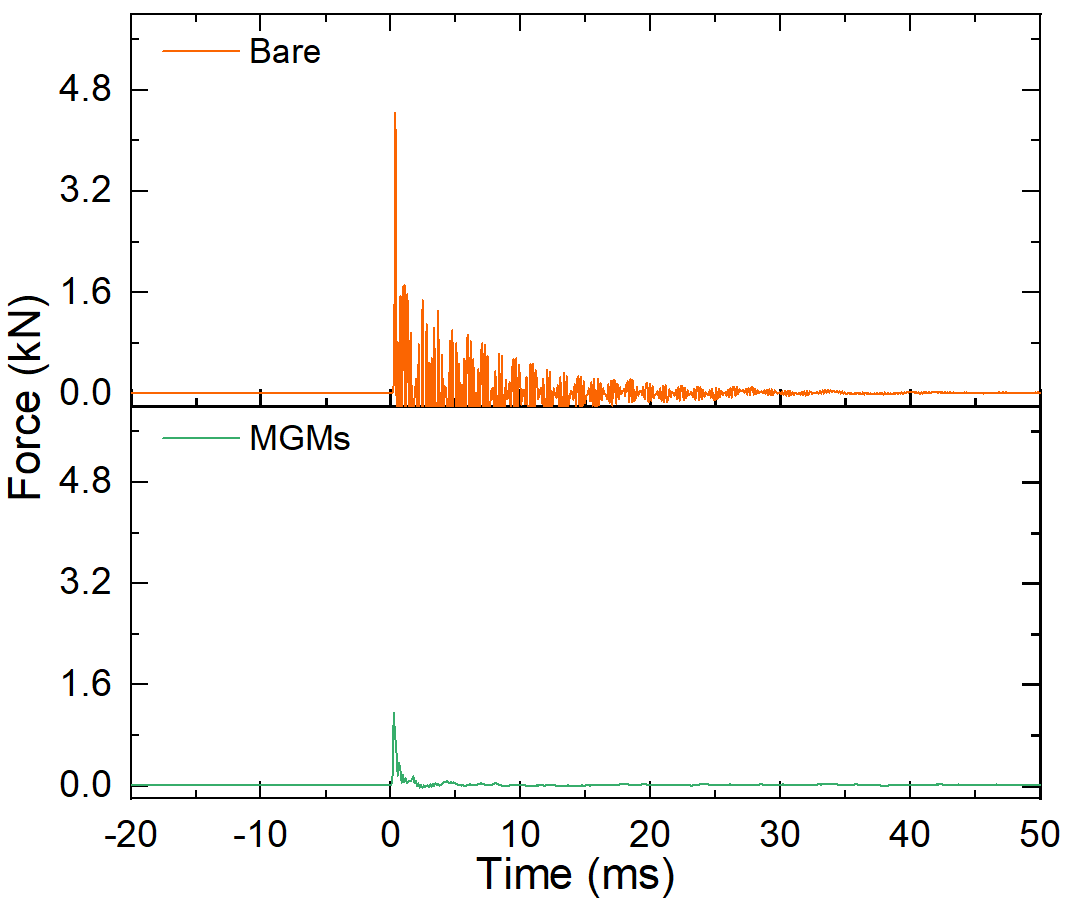


**Figure S40.** Force-time curves of impacted glass protected with nothing and MGMs coating. The MGMs coating has good force dissipation efficiency and buffering effect from the force-time curve.


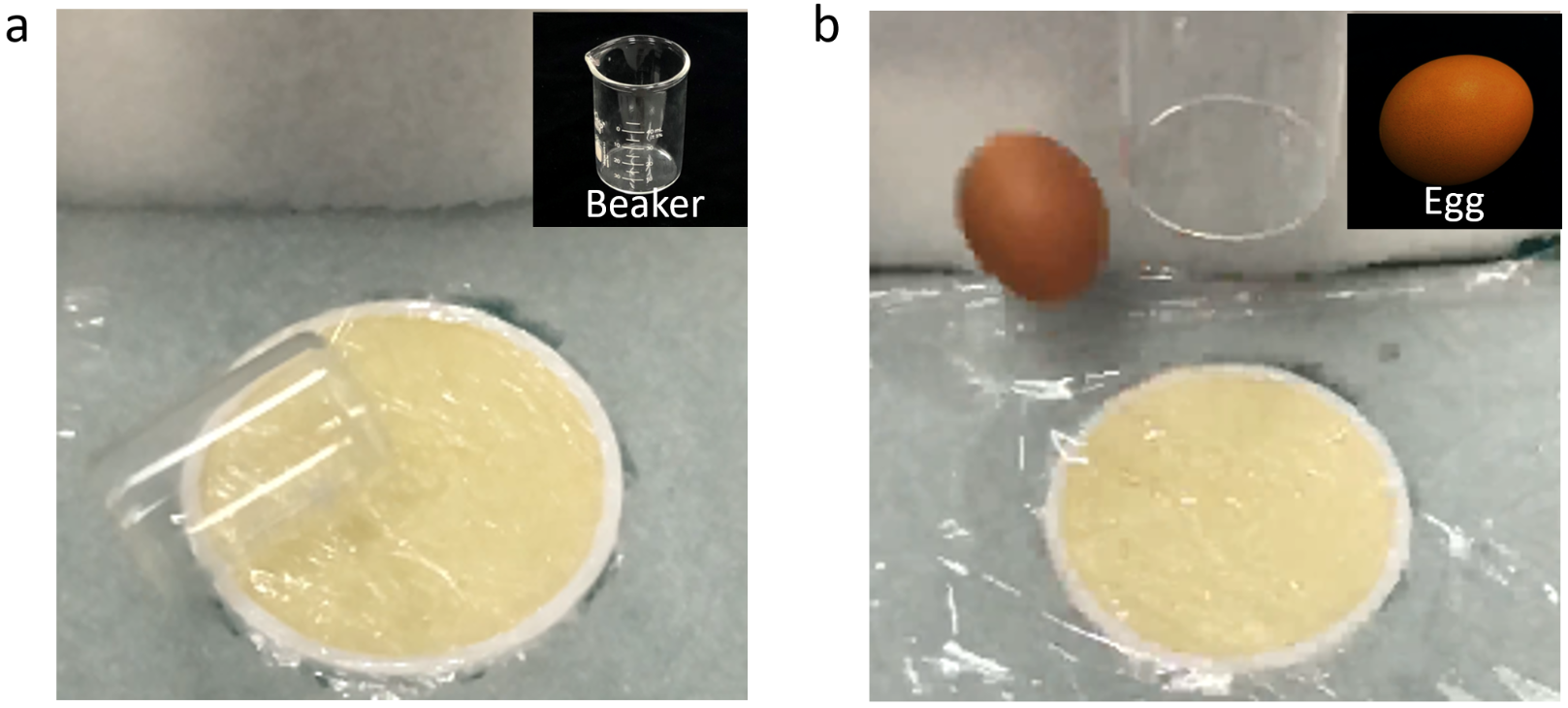


**Figure S41.** Snapshots of (a) beaker and (b) raw egg hitting MGMs that freely falling from 2.2 m and 1.2 m height, respectively. The thickness of MGMs sample is 5 mm.
